# FYN: emerging biological roles and potential therapeutic targets in cancer

**DOI:** 10.1186/s12967-023-03930-0

**Published:** 2023-02-05

**Authors:** SanFei Peng, Yang Fu

**Affiliations:** Department of Gastrointestinal Surgery, The First Affiliated Hospital of Zhengzhou University, Zhengzhou, 450052 China

**Keywords:** FYN, cancer, Targeted therapy, Biological functions

## Abstract

Src family protein kinases (SFKs) play a key role in cell adhesion, invasion, proliferation, survival, apoptosis, and angiogenesis during tumor development. In humans, SFKs consists of eight family members with similar structure and function. There is a high level of overexpression or hyperactivity of SFKs in tumor, and they play an important role in multiple signaling pathways involved in tumorigenesis. FYN is a member of the SFKs that regulate normal cellular processes. Additionally, FYN is highly expressed in many cancers and promotes cancer growth and metastasis through diverse biological functions such as cell growth, apoptosis, and motility migration, as well as the development of drug resistance in many tumors. Moreover, FYN is involved in the regulation of multiple cancer-related signaling pathways, including interactions with ERK, COX-2, STAT5, MET and AKT. FYN is therefore an attractive therapeutic target for various tumor types, and suppressing FYN can improve the prognosis and prolong the life of patients. The purpose of this review is to provide an overview of FYN’s structure, expression, upstream regulators, downstream substrate molecules, and biological functions in tumors.

## Introduction

Tyrosine Kinases(TK) consists of 90 enzymes whose main function is to catalyze the transfer of ATP phosphate groups to tyrosine residues of target proteins [1]. According to their structures, they can be divided into receptor protein tyrosine kinases (RTKs) and non-receptor protein tyrosine kinases (NRPTKs). RTKs include EGF receptor family, MET receptor family, ALK receptor family, FGF receptor family, RET receptor family, VEGF receptor family, Eph receptor family and DDR family etc. NRPTKs include SRC family, SYK family, FES family, FAK family, ABL1 and BCR-ABL family, and JAK family etc. The substrates are phosphorylated as a signaling mechanism between the cell surface, cytoplasmic proteins, and nuclear activation [2]. When cells are exposed to external and internal stimuli, TKs participate in cell proliferation, survival, differentiation, and metabolism [3, 4].

**Fig. 1:**
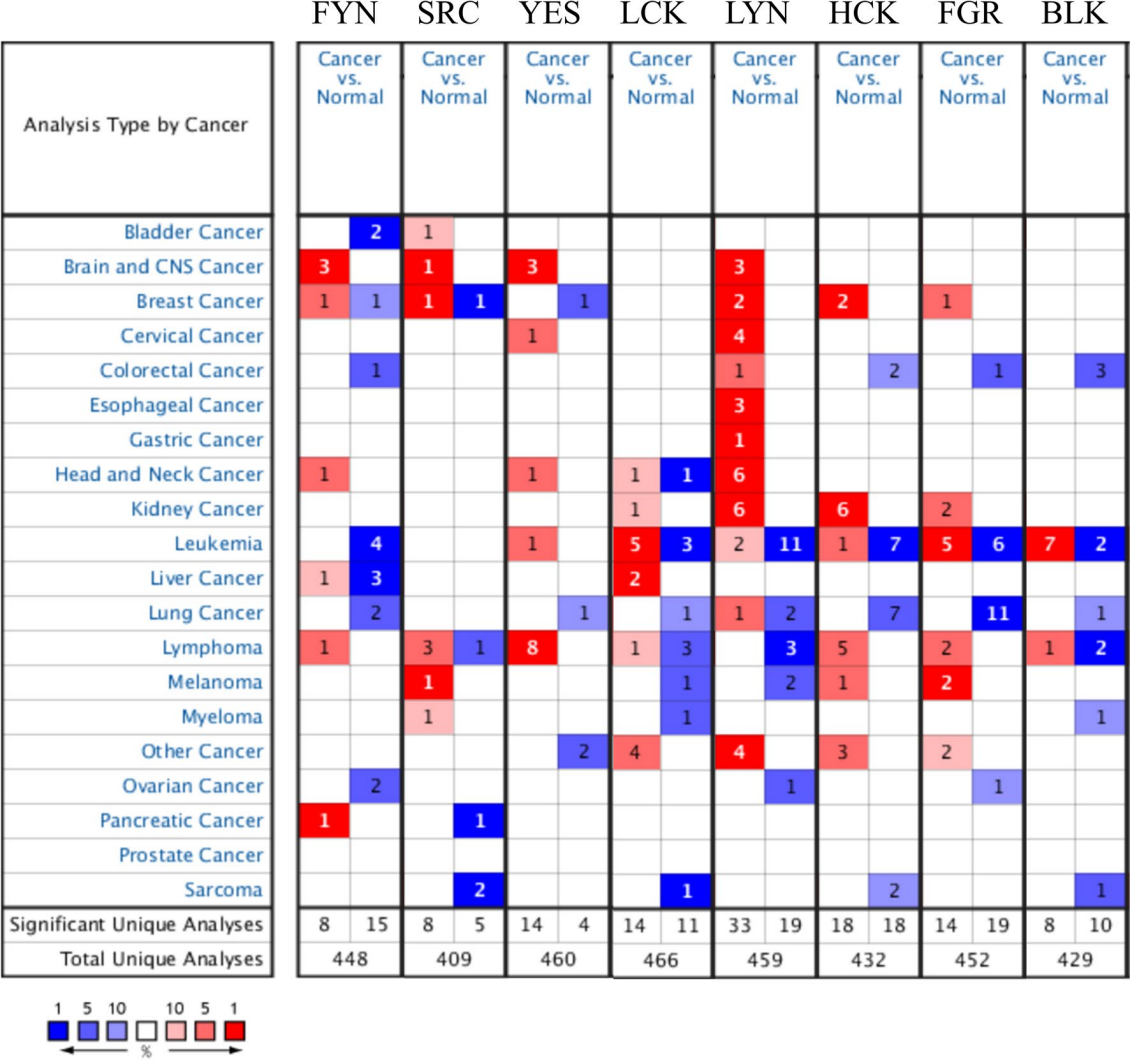
The mRNA expression levels of the SRC family in human cancers. The number of analyses that meet the thresholds was shown in the colored cells. The gene rank determines the cell color, which represents the importance of genes in cancer. The red and blue indicate over-expressed and under-expressed respectively, and the brighter red or blue implies a gene with a higher or lower level of expression that is more statistically significant

The SRC family of kinases (SFKs) is one of the overexpressed TKs in cancers. Previous studies identified eight SRC family kinases (SFKs) in Homo species, and several of these genes play crucial roles in cancer progression. The Oncomine platform contained 448 unique analyses for FYN expression, which was overexpressed in 8 of 448 unique analyses. SRC had significant expression in 8 of 409 unique analyses, YES had significant expression in 14 of 460 unique analyses, LCK had significant expression in 14 of 466 unique analyses, LYN had significant expression in 33 of 459 unique analyses, HCK had significant expression in 18 of 432 unique analyses, FGR had significant expression in 14 of 452 unique analyses, and BLK had significant expression in 8 of 429 unique analyses (Fig. 1).They have been proposed as molecular targets for treatment for decades [5]. FYN, also known as p59-FYN, SLK, SYN, is a 59 kDa protein containing 537 amino acids with genetic information located on chromosome 6q21 and was originally identified as a member of the SFKs [6]. FYN is primarily localized to cytoplasmic leaflets in the cytoplasmic membrane, which phosphorylates tyrosine residues of the key molecules involved in different signaling pathways [7]. FYN is a tyrosine kinase involved in transporting various cell surface receptors from the cytoplasmic signaling cascade. FYN contains the N-terminal region required for plasma membrane binding, and two Src homology (SH) domains (SH2 and SH3) are involved in protein interactions and are highly conserved catalytic domains, including the adenosine triphosphate (ATP) binding site and the C-terminal tail, which contains a negative regulatory tyrosine site phosphorylation [8]. In between the SH2 and SH1 structural domain is a circular SH2-linked mediator with a pseudo-SH3 binding site containing a tyrosine residue (Y416), which is activated by autophosphorylation and is required for its optimal activity [9, 10]. The active site of the kinase is in the SH1 structural domain, followed by the C-terminal regulatory fragment. Dephosphorylation of tyrosine residue (Y527) activates SKFs because it exposes the SH1 region tyrosine site, which can be modified by phosphorylation [11]. FYN regulates cell growth, survival, adhesion, cytoskeletal remodeling, motility, axon guidance, synaptic function, and central myelin-forming nervous system, in addition to platelet activation and T-cell receptor signaling [12–17].

## Regulatory mechanisms of FYN expression and activity

###  Protein levels significantly impact protein kinase activity, which in turn greatly influences the biological significance of protein kinases. Thus, the main factors affecting FYN expression in cancer are transcription factors, miRNA, and ubiquitinated degradation (Fig. 2)

**Fig. 2:**
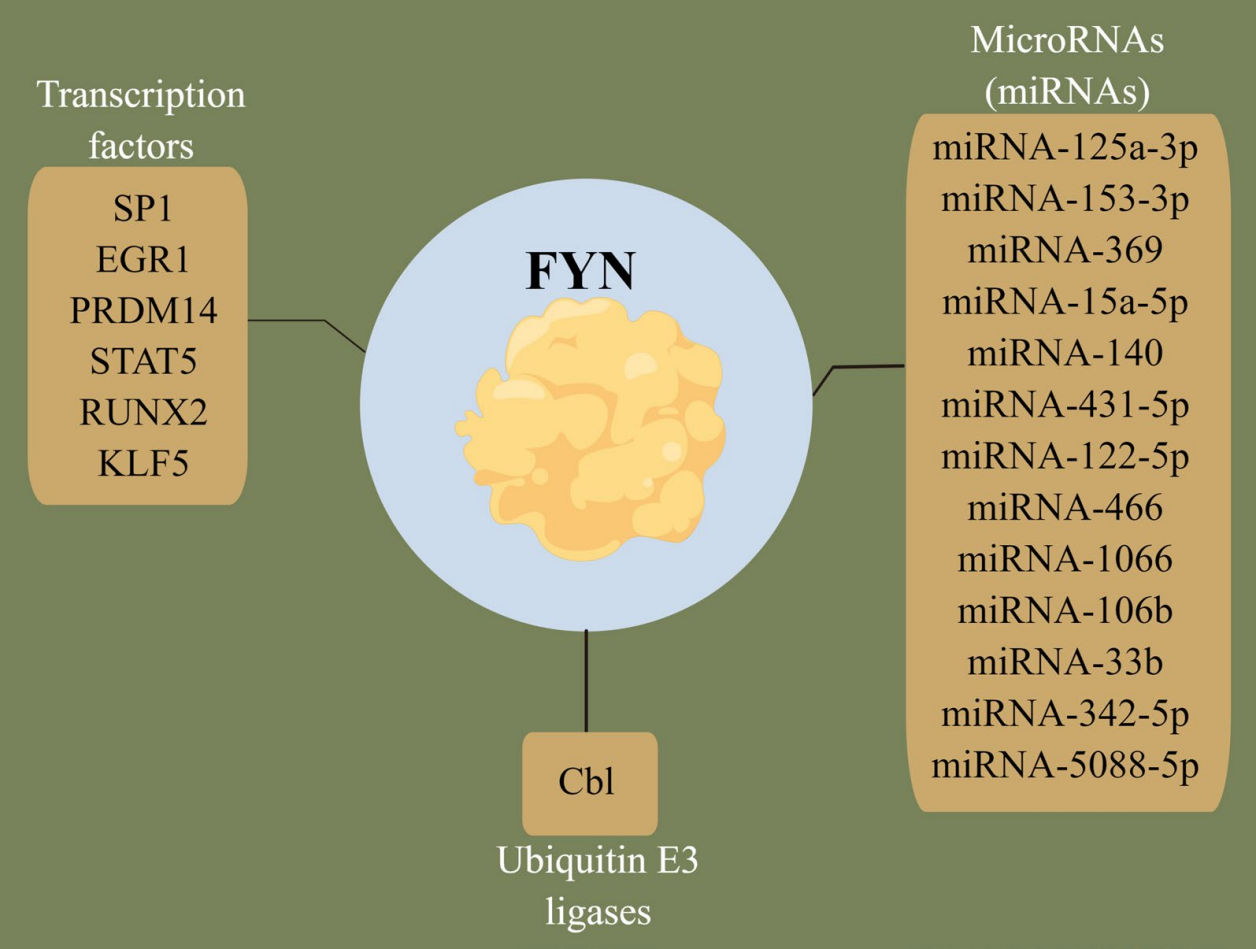
Major factors regulating FYN mRNA and protein expression. The main factors affecting FYN expression in cancer are transcription factors, miRNA, and ubiquitin E3 ligases

#### Transcription factors

During chronic myeloid lymphocytic leukemia (CML), the transcription factors SP1 and EGR1 bind to the FYN promoter, which reduces FYN expression [18]. FYN expression levels and downstream protein activation are decreased in pancreatic cancer cells where transcription factor PRDM14 is knocked down [19]. High levels of FYN and STAT5 are present in the positive feedback loop between basal breast cancer cells. FYN interacts directly with STAT5 and increases p-STAT5, which further acts as a transcription factor for FYN [20]. In Acute lymphoblastic leukemias, FYN is a target gene for the transcription factor RUNX2 [21]. Additionally, KLF5 binds to the FYN promoter region to induce its transcription, and overexpression of FYN improves lamellar pseudopod formation and migration in bladder cancer cells in which expression of KLF5 is reduced [22].

#### MicroRNA (miRNA)

It has been demonstrated that miR-125a-3p regulates FYN expression and contributes to the progression of multiple cancers [23–28]. FYN is a downstream target of miR-153-3p, and the downregulation of miR-153-3p levels promotes FYN expression for esophageal squamous cell carcinoma (ESCC) proliferation [29]. The miR-381 inhibits MAPK signaling by downregulating FYN, thereby making breast cancer cells more sensitive to doxorubicin (DOX) [30]. miR-369 also has been demonstrated to target the 3’UTR of FYN to regulate its expression [31]. Several proteins in the megakaryocyte GPVI signaling pathway, including FYN, are regulated by miR-15a-5p [32]. miR-140 inhibits FYN kinase mRNA to establish axon-dendritic polarity [33]. FYN is regulated by miR-431-5p in diffuse large B-cell lymphoma (DLBCL) [34]. Bioinformatic analysis revealed that FYN is a target gene of miR-122-5p[35]. miR-466 overexpression significantly reduces the expression of a network of transcription factor RUNX2 target genes, including FYN, and inhibits tumor growth and bone metastasis in prostate cancer [36]. A systematic analysis of miRNA-mediated gene regulation in tamoxifen-resistant breast cancer cell lines (TamRs) compared to their parental tamoxifen-sensitive cell lines demonstrated that miR-33b, miR-342-5p were significantly associated with FYN and can directly regulate its expression [37]. FYN can also promote the biosynthesis of miR-5088-5p by inducing its hypermethylation to mediate breast cancer proliferation and metastasis [38]. In the current study, several miRNAs were identified that could regulate FYN expression-mediated role in cancer. More miRNAs associated with FYN expression will likely be discovered in the future.

#### Degradation of post-translational ubiquitination

It has been demonstrated that endogenous Cbl mediates the ubiquitination of FYN and determines the rate of FYN protein turnover [39, 40].

### The upstream regulators of FYN

The upstream regulators of FYN

**Fig. 3:**
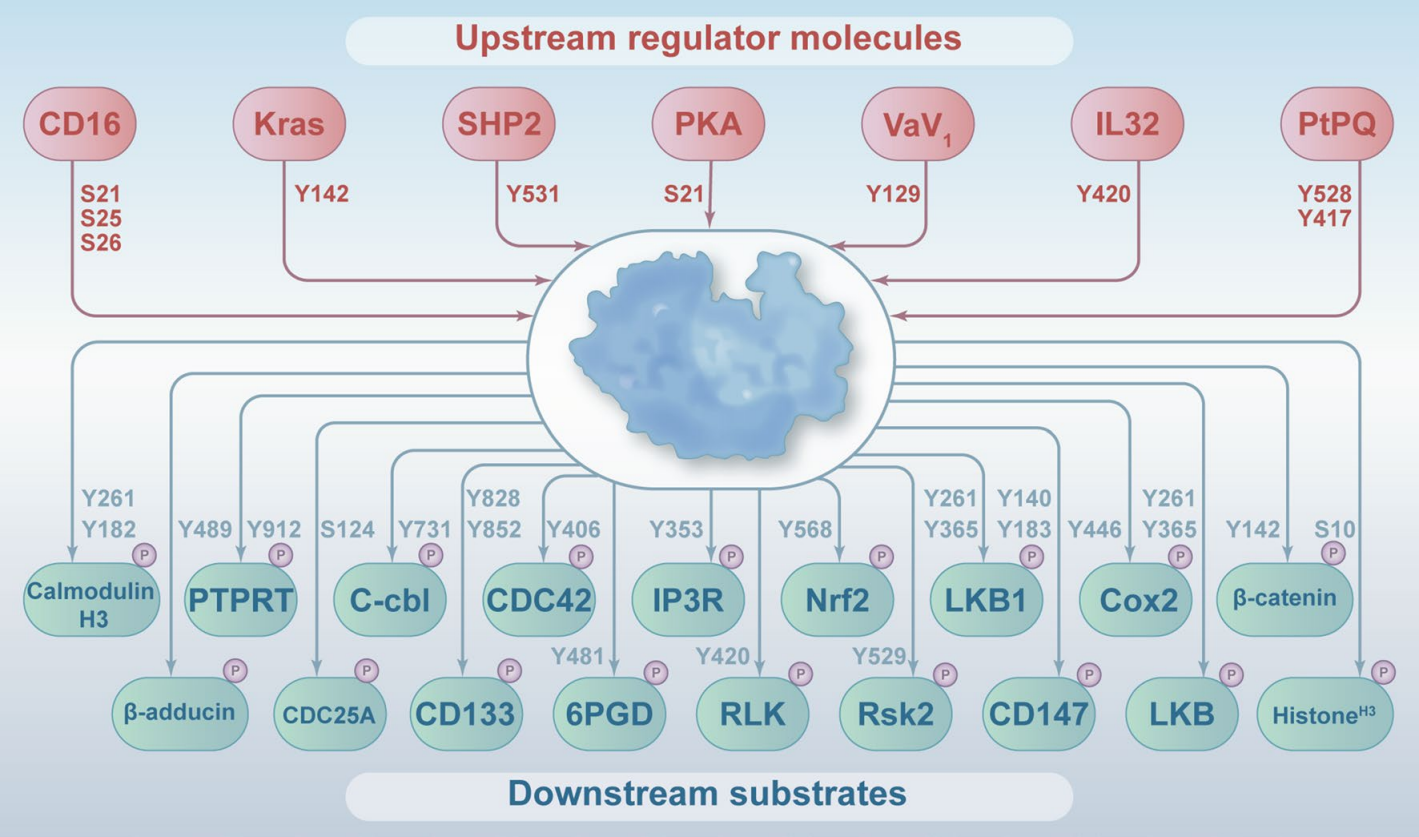
An overview of FYN-interacting proteins. Multiple molecules can regulate FYN activation or inactivation through phosphorylation, and once activated, FYN interacts with and phosphorylates a wide variety of proteins serving as mitotic regulators, oncogenes or tumor suppressors

The function of FYN as a protein kinase in cancer is regulated by protein levels and its activity, and this section will focus on its upstream regulatory molecules (Fig. 3).

By activating FYN, Integrin beta6 can trigger the Raf-ERK/MAPK pathway to promote the progression of oral cancer [41]. SHP2 facilitates the localization and activation of FYN downstream of alpha6beta4 integrin to promote cancer invasion [42]. According to Akash Gulyani and colleagues, adhesion/integrin signaling influences cellular FYN activity [43]. Expressing constitutively active EGFR mutant EGFRvIII results in FYN phosphorylation, which promotes glioblastoma progression and invasion [44]. In breast cancer, the Ras oncogene significantly upregulates FYN mRNA, protein, and kinase activity [45]. The binding of platelet-derived growth factor (PDGF) to its receptor leads to activation of the protein tyrosine kinase FYN, which is phosphorylated on the N-terminal portion of Tyr28 after interacting with the intracellular structural domain of the PDGF β receptor. Subsequently, it is autophosphorylated on Tyr30, Tyr39 and Tyr420 [46]. It has been displayed that FYN is phosphorylated by PKA on S21. This phosphorylation regulates FYN activity, adherent spot targeting and is required for cell migration, and mutating S21 to S21A blocks PKA-mediated FYN phosphorylation and alters its tyrosine kinase activity [47]. Kinome analysis of human natural killer cell receptor-induced phosphorylation revealed that triggering CD16 resulted in phosphorylation of FYN at N-terminal S21, S25, and S26, while adjacent Y28 depicted a trend towards dephosphorylation [48]. In the FYN/ Vav1 complex, FYN can be phosphorylated at the Tyr-129 [49, 50]. FYN kinase activity is inhibited by PTPa knockdown, and elevated FYN activity in the presence of PTPa results from increased phosphorylation of FYN at Tyr-528 and Tyr-417 [51]. CD5 activation induced tyrosine phosphorylation of FYN and inhibited phosphorylation of ZAP-70, and FYN activity was inhibited by phosphorylating its inhibitory C-terminal Tyr531 [52]. IEC18 cells transfected with K-ras have increased phosphorylation of FYN on Tyr-142 and elevated kinase activity than controls [53]. SHP2 knockout cells demonstrated increased phosphorylation and reduced kinase activity at the inhibitory pY531 of FYN [54]. LL-37 and IG-19 inhibit IL-32/FYN kinase activity by inhibiting the Y420 of FYN phosphorylation mediated by interleukin-32 (IL-32) [55]. CD5-FYN phosphorylation is maintained at equilibrium and controlled by the kinase activity of FYN, which phosphorylates PAG-Tyr317. This phosphorylation allows docking of Csk, which in turn phosphorylates FYN at its C-terminal inhibitory tyrosine, leading to a decrease in FYN activity and thus closing this activation loop [52, 56, 57]. Integrin-mediated hyperphosphorylation of FYN activation and new protein synthesis was observed after stimulation in highly metastatic cells [58]. By activating FYN, HGF/MET promotes the progression of prostate cancer [59, 60]. Both classical Wnt3a and non-classical Wnt5a pathways stimulate Fz2 phosphorylation, FYN activation by Fz2, and FYN-dependent phosphorylation of Stat3 [61]. Hyperphosphorylation of autophosphorylation site tyrosine in FYN was detected in protein tyrosine phosphatase N23 (PTPN23)-deficient breast cancer tumors, confirming that FYN could be a therapeutic target for PTPN23 heterozygous or pure deletion breast tumors [62]. Integrins are involved in triggering FAK-Y397 phosphorylation, and a portion of FAK is located in the lipid raft/fossa structural domain where it interacts with FYN leading to elevated levels of FYN phosphorylation and elevated activity [63]. Microarray transcriptomic and bioinformatic analysis of ovarian cancer identified FYN as a key downstream target in the transcriptome of GNAi2/gip2 regulated tumor progression [64].

## The downstream substrates regulated by FYN in tumors and its biologic functions

###  Substrates regulated by FYN (Fig. 3)

FYN phosphorylates calmodulin h3 and calmodulin h1 at the Tyr261 and Tyr182 [65]. FYN was also found to phosphorylate AMPKa at Y436 and inhibit its enzymatic activity without affecting the assembly of the heterotrimer complex of AMPK [66]. FYN phosphorylates the IP(3) receptor at the Tyr 353 [67]. One study demonstrated that FYN phosphorylates β-adducin at Tyr-489, located in its C-terminal tail structural domain [68]. The phosphorylation of Sam68 by FYN reverses this action and facilitates the selection of the Bcl-x(L) splicing site [69]. Phosphorylation of Nrf at Tyr-568 by FYN results in nuclear export of Nrf2, binding to Nrf2, and degradation of Nrf2 [70, 71]. The selective regulation of Pyk2 phosphorylation by FYN in vivo correlates with FYN’s preferential phosphorylation of Pyk2 in vitro [72]. FYN phosphorylates AMPK to inhibit AMPK activity and AMP-dependent activation of autophagy, and in addition, FYN directly phosphorylates LKB1 at Y261 and 365, and mutations at these sites result in LKB1 export to the cytoplasm and increase AMPK phosphorylation [66, 73]. 32^P^ radiolabeled in vitro kinase assays displayed phosphorylation of COX2 by FYN, and further studies revealed that phosphorylation of residue Y446 in the COX2 enzyme by FYN resulted in increased enzyme activity without altering the protein level of COX2, which is a direct substrate for phosphorylation by FYN. FYN constitutively associates with and phosphorylates Cas, suggesting that tyrosine phosphorylation of Cas may be catalyzed by FYN [74–76]. The Tyr-828 and Tyr-852 sites of the stem cell marker CD133 are phosphorylated in the cytoplasm by FYN tyrosine kinase [77]. It has been demonstrated that increased tyrosine phosphorylation of GSK-3beta directly corresponds to the increased association of FYN, suggesting that FYN may phosphorylate GSK-3beta or mediate phosphorylation of GSK-3beta [78]. Activated FYN kinase phosphorylates histone H3 at Ser-10 [79]. FYN phosphorylates IP3R1 in Tyr353[80]. Phosphorylation of IFITM3 by FYN leads to a decrease in IFITM3 ubiquitination [81]. FYN kinase directly phosphorylates LKB1 at Y261 and Y365, and mutations at these sites result in LKB1 export to the cytoplasm and increased AMPK phosphorylation [73, 82, 83]. FYN phosphorylates Nrf2 Y568, leading to nuclear export and degradation of Nrf2 [84, 85]. Y420 is a major site of phosphorylation of RLK by FYN, and phosphorylation of this site activates RLK kinase [86]. In an in vitro kinase assay, Src and FYN were able to phosphorylate RSK2 directly at Tyr-529 [87].The differential potential of FYN to phosphorylate Sam68 can be controlled by the interaction of the kinase SH3 structural domain with the linker and Sam68, possibly based on competitive binding [88]. FYN phosphorylates and activates ZAP-70, two kinases that cooperate in TCR signaling [89]. FYN drives G6PD by phosphorylating STAT3 expression, leading to the promotion of tumor growth and inhibition of cellular senescence [90]. It has been demonstrated that FYN directly phosphorylates CD147 at Y140 and Y183, while CD147-FF (Y140F/Y183F) mutation impairs the interaction between CD147 and FYN, and knockdown of FYN expression significantly attenuates the malignant phenotype of melanoma cells by downregulating CD147 phosphorylation [91, 92]. FYN interacts with ARHGEF16 to regulate the proliferation and migration of colon cancer cells, and knockdown of FYN expression decreases ARHGEF16 protein levels in colon cancer cells[93]. Inhibition of FYN blocks the phosphorylation level of FAK/N-WASP, which in turn prevents hepatic stellate cell (HSC) activation, proliferation, and migration[94]. In breast cancer cells, FYN knockdown led to reduced phosphorylation of zeta/delta (Thr232) and Cdc25A (Ser124) [95]. Upon EGFR activation, 6PGD is phosphorylated by FYN at tyrosine Y481, and this phosphorylation enhances 6PGD activity, which activates NADPH and PPP of ribose 5-phosphate, thereby detoxifying intracellular reactive oxygen species (ROS) and accelerating DNA synthesis [96]. Inhibition of FYN activity in pancreatic cancer is upregulated by P21-activated kinase 1 expression and promotes phosphorylation and nuclear localization of hnRNP E1, leading to the construction of a spliceosome complex that affects variable splicing of integrin β1 [97]. There is negative reciprocal regulation between SMAD4 and FYN in ovarian tumors, and knockdown of SMAD4 results in elevated levels of FYN expression, and FYN activation leads to dissociation of cell-cell junctions and adhesion, resulting in increased tumor metastasis [98]. FYN affects proliferation, apoptosis, migration, and invasion of pancreatic cancer cells through phosphorylation of GluN2b and regulation of the AKT signaling pathway [99]. In angio-immunoblastogenic T-cell lymphoma (AITL) and peripheral T-cell lymphoma not otherwise specified (PTCL, NOS), the FYN-TRAF3IP2 fusion gene induces aberrant NF-κB signaling downstream of T-cell receptor activation, and inhibition of FYN-TRAF3IP2-induced NF-κB signaling in tumors with an IκB kinase inhibitor provides potent anti-lymphoma effect [100].

### Biological functions of FYN kinase in cancer

**Fig. 4:**
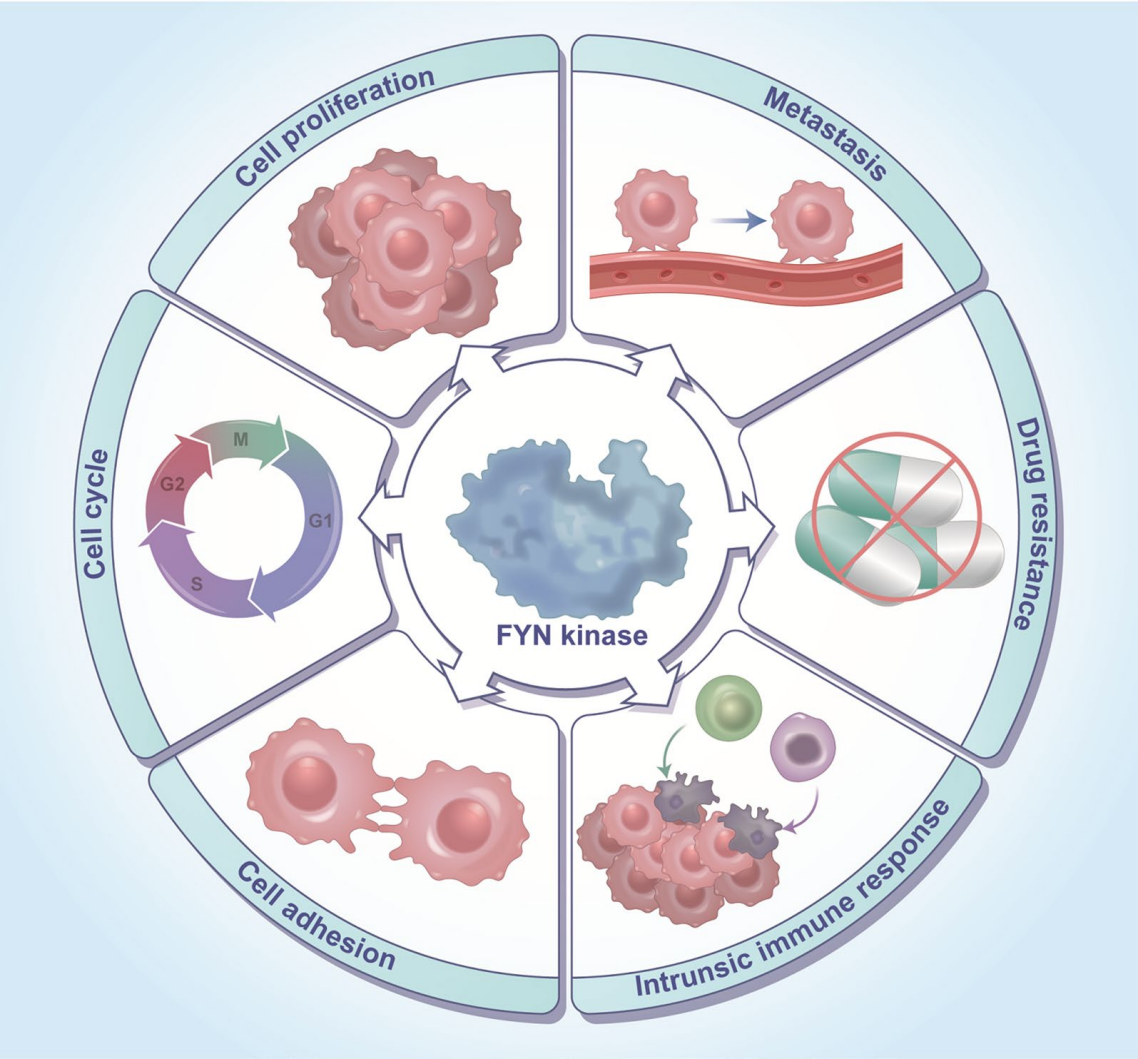
Multiple biological functions of FYN in cancer. Mainly includes the role of FYN in cell cycle, cell adhesion, proliferation, metastasis, drug resistance, and intrinsic immunity

Current evidence indicates that FYN plays a pro-oncogenic role in cancer development. The role of FYN in cell cycle, cell adhesion, proliferation, metastasis, drug resistance, and intrinsic immunity will be discussed in this section(Fig. 4).

####  FYN regulates the tumor cell cycle

FYN functions as a member of the Src family of kinases to prevent cytoplasmic division after mitosis utilizing anti-SH2, and as a result, cell division is inhibited [101, 102]. It has been demonstrated that FYN regulates mitotic spindle formation through its effect on microtubule polymerization and stabilization.FYN promotes mitotic spindle formation through increased microtubule aggregation, leading to cell formation that accelerates M-phase progression [103]. Insufficient FYN activity has been reported to cause cytoplasmic division failure and prevent mitosis from proceeding [104]. During cytoplasmic division, FYN is localized to the cortical membrane-bound domain depending on its N-terminal length [105]. Cortical FYN is thought to be involved in regulating cytoplasmic division [104]. The above findings suggest that FYN inhibits mitotic progression and blocks pericellular progression.

####  FYN regulates tumor cell adhesion

Intercellular adhesion was enhanced by inhibiting FYN activity with dasatinib or silencing FYN [106]. The initial adhesion of T cells follows activation, but it does not require the action of FYN kinase. However, in late cell adhesion, non-catalytically functional FYN is required [107]. Phosphorylated FYN (pTyr530) is upregulated in Integrin α6-deficient acute lymphoblastic leukemia (ALL) and mediates the development of chemoresistance through adhesion [108].

####  FYN regulates tumor cell proliferation

FYN is a proto-oncogene belonging to the Src family, which has been reported in many studies to promote cancer cell proliferation and inhibit apoptosis. FYN is an important mediator and regulator of mitogenic signaling cell cycle entry, growth, and proliferation [60]. FYN is upregulated in thyroid cancer at both mRNA and protein expression levels, which promotes cell proliferation and inhibits apoptosis in thyroid cancer [109]. FYN is a direct target of microRNA-125a-3p, which directly inhibits the expression and activity of FYN, and induces cell cycle capture and expression of FYN downstream proteins, which in turn inhibits cell proliferation. This suggests that FYN promotes tumor cell proliferation [23]. In chronic granulocytic leukemia, increased FYN expression and activity promote the transition from chronic granulocytic leukemia to the acute phase and accelerate cell proliferation [110]. FYN induces osteoclast proliferation inhibiting osteoclast apoptosis [111]. FYN expression is dysregulated in acute myeloid leukemia (AML) patient samples, and FYN is associated with wild-type FLT3 and oncogenic FLT3-ITD. This correlation depended on the kinase activity of FLT3 and the SH2 structural domain of FYN. Multiple FYN binding sites were present in FLT3, and FYN expression induced slightly enhanced phosphorylation of AKT, ERK1/2, and p38 and effectively enhanced STAT5 phosphorylation and colony formation. Moreover, higher expression of FYN in combination with FLT3-ITD mutation resulted in enrichment of the STAT5 signaling pathway and was associated with poor prognosis in AML. These results demonstrate that FYN promotes AML cell proliferation by selectively activating the STAT5 pathway in cooperation with oncogenic FLT3-ITD in cell transformation [112]. LINC00152 promotes esophageal squamous cell carcinoma (ESCC) proliferation by downregulating miR-153-3p and promoting FYN expression [29]. In glioblastoma, FYN phosphorylates PIKE-A and thus promotes its binding to AMPK, inhibits the tumor suppressive effect of AMPK, and promotes tumor cell proliferation [113]. Inhibition of FYN activity inhibits pancreatic cancer cell proliferation [114]. Skin squamous cell carcinoma (SCC) cells, increased FYN activity decreases Notch1/NICD mRNA and protein expression levels and promotes STAT3 phosphorylation to induce proliferation and tumorigenesis [115]. FYN phosphorylates STAT3 and promotes G6PD expression, promoting malignant glioma growth and inhibiting cellular senescence [90]. FYN interacts with ARHGEF16 to promote colon cancer cell proliferation [93]. The FYN/STAT3 pathway inhibits melanoma cell growth [116]. FYN stimulates pancreatic cancer progression through phosphorylation of GluN2b and the regulated AKT protein kinase signaling pathway [99]. 5 ‘nucleotidase domain containing 2 (NT5DC2) promotes glioblastoma progression by upregulating FYN expression levels [117].

#### FYN regulates tumor epithelial-mesenchymal transition (EMT) and metastasis

Epithelial-mesenchymal transition (EMT) is the transformation of acute epithelial cells into adjacent mesenchymal cells that occurs during embryonic transdifferentiation and plays an extremely important role in cancer metastasis [118, 119]. FYN upregulates the expression of mesenchymal markers of breast cancer, epithelial-mesenchymal transition (EMT)-related transcription factors, and downregulates the expression of epithelial cells to induce the development of EMT [120]. It has been revealed that epithelial integrin αvβ6 complexes with FYN kinase in oral SCC promote EMT and migration [121]. miR-125a-3p inhibits epithelial-mesenchymal transition in pancreatic ductal adenocarcinoma (PDAC) by directly targeting FYN [26]. FYN has also been demonstrated to promote EMT and tumor metastasis in colon cancer cells [122].It has been demonstrated that FYN promotes cell migration and invasion by regulating the AMPK / mTOR signaling pathway in CCA cell lines, and therefore knocking down FYN expression levels is an effective option for anti-CCA therapy [123]. In thyroid cancer, FYN can also control tumor cell migration and invasion [109]. The expression of FYN by KLF5 can increase tumor invasion and cell migration in bladder cancer[22]. The DEP-1-FYN reciprocal regulatory loop section promotes the migration of microglia in brain tissue [124]. Ras/PI3K/Akt signaling can increase FYN overexpression in cancer, thereby promoting tumor cell migration and invasion [45].FYN forms a molecular complex with Nck and PAK-2 and p-Tyr molecular complexes with Nck and PAK-2 and assembles in a p-Tyr1214-dependent manner. This triggers activation of the SAPK2/p38 MAP kinase module and promotes endothelial cell migration [125]. Chemoattractant receptor binding induces FYN-dependent PI3K activation bound to LFA-1 and suggests that FYN is required to initiate and/or regulate chemoattractant-mediated LFA-1 activation to promote targeted migration [126]. Inhibition of FYN activity inhibits metastasis in pancreatic cancer [114]. FYN and its downstream molecular signaling pathway proteins are upregulated in prostate cancer expression [127]. FYN promotes the maintenance of the neuroendocrine phenotype of tumor cells in progressive prostate cancer as well as Vascular metastasis of cancer [60]. miR-125a-3p overexpression inhibits the activity of FYN, FAK, and paxillin, thus suppressing prostate cancer metastasis [27]. FYN is recruited to the α6β4/ SHP2 complex by interaction with phospho-Y580 at the C-terminus of SHP2 for activation, and this Y580-SHP2 interaction localizes FYN to the receptor binding site, which is required for α6β4-dependent promotion of invasive metastasis [42]. It has been demonstrated that upregulation of FYN expression is associated with metastasis in human pancreatic cancer. Inhibition of FYN activation by kinase-inactivating FYN transfection in BxPC3 pancreatic cancer cells reduced liver metastasis in nude mice [128]. At this stage, we have revealed that FYN in gastric cancer promotes proliferation and metastasis through phosphorylation of TOPK to enhance its oncogenic activity and activation of TOPK downstream proliferation and metastasis-related signaling pathways. FYN and ARHGEF16 interact to promote the migration of colon cancer cells [93]. Ampelopsins A and C induce cell metastasis by downregulating FYN expression in breast cancer cells [129]. IBSP promotes the growth and invasiveness of colorectal cancer (CRC) by a potential mechanism of activating the FYN/β-catenin signaling pathway [130]. FYN expression is elevated in melanoma cells, and knockdown of FYN significantly inhibits the proliferation and migration of melanoma cells by downregulating CD147 phosphorylation [92]. FYN has been demonstrated to promote gastric cancer metastasis by activating STAT3-mediated epithelial-mesenchymal transition [131]. A TCGA cancer database analysis based on genes related to lipid metabolism in colon adenocarcinoma found that FYN gene expression was associated with the activation of the EMT pathway [132]. It has been shown in another study that increased FYN expression may contribute to hepatocellular carcinoma metastasis [133].The Wnt5-Fzd2-FYN-Stat3 axis contributes to the EMT program, cell migration, and multiple tumor metastases, and the FYN inhibitor Dasatinib inhibits this process [122].

#### The role of FYN in tumor drug resistance

The emergence of drug resistance remains a formidable challenge for the effective treatment of cancer patients, and several studies have found that FYN promotes drug resistance in tumors. Knockdown of FYN protein expression levels rather than inhibition of its activity sensitizes TKI-resistant cells to dasatinib, a dual BCR-ABL1/Src inhibitor [134]. Knockdown of FYN kinase by pharmacological inhibition or siRNA-mediated re-sensitization of chronic granulocytic leukemia (CML) cells to the BCR-ABL inhibitor imatinib-resistant cell line (IM-R cells) to imatinib [135]. Concurrently, FYN overexpression in the tamoxifen-sensitive group reduced sensitivity to tamoxifen treatment. At the same time, knockdown of FYN expression restored sensitivity to tamoxifen, and mechanistic studies suggested that FYN overcomes the antiproliferative effects of tamoxifen by activating important cell cycle-related proteins [95]. miR-381 promotes the chemosensitivity of breast cancer cells to DOX by downregulating FYN to inactivate MAPK signaling[30]. Overactivation of FYN in dasatinib-resistant cell promotes the development of drug resistance [136]. FYN causes tamoxifen resistance in breast cancer (ER+), and knockdown of FYN expression or use of FYN inhibitors significantly inhibits the growth of tamoxifen-resistant cells and the association with poor prognosis in breast cancer [37]. The involvement of FYN in anticancer drug resistance has been demonstrated, where increased FYN expression was associated with resistance to imatinib in the K562 cell [137]. FYN modulates imatinib resistance in prostate cancer patients through interaction with miR-128/193a-5p/494 [138]. Thus, FYN is highly expressed in several cancer-resistant cell and is involved in developing cancer drug resistance.

#### FYN and intrinsic immune response

The FYN splice variant (FYNT) was first identified in T lymphocytes, and the development and activation of lymphocytes, macrophages, dendritic cells, and natural killer (NK) cells is enhanced by increased expression or activation of Src and its downstream protein PI3K [12, 17, 139, 140]. One study confirmed that antigen-specific T cell activation depends on FYN activity and its knockdown severely impairs T cell responses [140]. Clinical studies in CML patients treated with dasatinib (a Bcr-Abl tyrosine kinase inhibitor that also inhibits SFKs) revealed transient immunosuppression characterized by IgE-dependent activation of hemophilic and T-cell receptor-dependent activation of T lymphocytes [141]. Another study demonstrated SFK inhibitor effects on patients after dasatinib treatment. The loss of FYN binding to intraluminal leaflets accompanied by lipid perturbation attenuated NK cell activation [142]. FasL overexpression enhances NK and T cell-mediated killing by recruiting FYN through proline-rich domains [143]. FYN in High expression of FYN in glioma cells reduces immune activation against glioma, and inhibition of FYN improves the efficacy of anti-glioma immunotherapy [144]. The FYN-ADAP pathway preferentially regulates cytokine production in NK and T cells [145]. FYN directly binds and phosphorylates ADAP, SKAP55, and SHP-2, while SHP-2 interacts with PD-1 to induce PD-1 + CTLA-4 + CD8 + TIL in tumors [146].

## The value of targeted FYN inhibition in cancer therapy

Several compounds have been shown to inhibit the kinase activity of FYN in cancer and have also shown to be of great value in cancer therapy. Most of these compounds are SRC family kinase inhibitors, and some of them also target other kinases, however, there is sufficient evidence to confirm that they may be valuable targets in clinical therapy (Table 1).

**Table 1:** Clinical trials in the context of SFKs. Data collected from clinicaltrials.gov on 10th Jan 2023

Inhibitor/Drug	Condition(s)	Phase	Clinical Trials ID	Refs
		of trial		
Saracatinib	prostate cancer	II	NCT01267266	[147]
Dasatinib	melanoma	II	NCT00700882	[148]
JNJ-26483327	solid tumors	I	NCT00676299	[149]
TPX-0046	Non Small Cell Lung Cancer	I, II	NCT04161391	NA
	Medullary Thyroid Cancer			
	RET Gene Mutation Metastatic Solid Tumor			
	Advanced Solid Tumors			
AZD0424	Advanced Solid Tumors	I	NCT01668550	[150]
Saracatinib	Small Cell Lung Cancer	II	NCT00528645	[151]
TPX-0022	Advanced NSCLC, Gastric Cancer or Solid Tumors	I, II	NCT03993873	NA
AZD0530	Non Small Cell Lung Cancer, Epithelial Ovarian Cancer	I	NCT01000896	NA
Ponatinib	Acute Lymphoblastic Leukemia	II	NCT05306301	NA
Bosutinib	Advanced Breast Cancer	I	NCT03854903	NA
KX2-391	Bone-Metastatic, Castration-Resistant Prostate Cancer	II	NCT01074138	[152]
Repotrectinib	Locally Advanced Solid Tumors/Metastatic Solid Tumors	I, II	NCT03093116	[153]
ON123300	Solid Tumors	I	NCT04739293	NA

## SFKs inhibitors in clinical studies

Several highly specific FYN inhibitors have been developed and shown to be effective in clinical trials. These inhibitors include mainly Dasatinib, Saracatinib, etc. In the following section, we elaborate on the role that these inhibitors play in cancer treatment.

**Saracatinib** is a highly specific small molecule inhibitor of SRC family kinases with an IC50 value of 10 nm against FYN. In a phase II clinical trial, Saracatinib was confirmed to act as a metastasis suppressor for prostate cancer in the initial stages [147].The anticancer drug saracatinib inhibits phosphorylation of invasion-associated substrates by inhibiting the SRC kinase, which results in a reduced invasive capacity for head and neck squamous cell carcinomas (HNSCC) [154]. Combined with 5-FU, saracatinib has also been shown to have enhanced antitumor effects in gastric cancer [155].It has shown a strong antitumor effect in preclinical models of Biliary tract carcinoma (BTC) [156]. Additionally, saracatinib can be used by itself or in combination with radiotherapy to treat malignant tumors, such as glioblastoma (GBM) [157].

**Dasatinib** is a novel and effective multitargeted inhibitor of kinases of the SRC family, as well as several other kinases. In a phase II clinical trial in melanoma, dasatinib was not significantly effective due to poor patient tolerance and dosage reductions in the study [148]. An immunotherapy-plus-dasatinib treatment of mice with liver metastases from colorectal cancer significantly increased immune cell infiltration into the tumor, therefore enhancing anti-tumor immunity [158]. Chemotherapy combined with dasatinib is also significantly more effective in treating tumors than chemotherapy alone [159–161]. As a result of its inhibition of multiple targets, dasatinib produces anti-growth, anti-angiogenic, and pro-apoptotic effects in oral cancer [162]. Dasatinib has also been shown to be effective in breast cancer [163, 164]. According to another clinical trial, Dasatinib inhibits T-cell receptor signaling and is therapeutic for Angioimmunoblastic T-cell Lymphoma [165]. An exploratory study in melanoma cell lines found that Dasatinib is anti-proliferative and anti-metastatic, and its combination with chemotherapy may improve responses [166].

**Ponatinib** is a multitarget inhibitor that targets primarily on Abl, PDGFRα, VEGFR2, FGFR1 and SRC, with an IC50 of 5.4 nM for SRC. It was found that chemotherapy and ponatinib together achieved early and sustained remissions for newly diagnosed Philadelphia chromosome-positive acute lymphoblastic leukaemia and improved the prognosis for patients [167].Another study of Philadelphia chromosome-positive leukemias also demonstrated significant anti-leukemic activity of ponatinib in terms of disease stage and mutation status [168]. Additionally to its therapeutic effects in CML and Ph + ALL, ponatinib has shown significant antitumor effects in some solid tumors [169, 170].

**Bosutinib** is a novel dual SRC/Abl inhibitor with an IC50 of 1.2 nM against SRC. A phase 4 clinical trial found that Bosutinib was more effective than TKIs for patients with Ph + CP CML [171, 172]. Bosutinib combined with Pemetrexed would be significantly more effective than either agent alone in metastatic solid tumors [173]. According to another clinical study, bosutinib in combination with chemotherapy significantly enhances antitumor activity in locally advanced or metastatic breast cancer [174]. Bosutinib inhibits the activation of EGFR, therefore reducing the progression of head and neck cancer [175]. In HeLa Cells,Bosutinib produces tumor suppressive effects by inhibiting Src/NF-κB/Survivin expression [176]. Bosutinib also inhibited the proliferation and migration of non-small cell lung cancer (NSCLC) in another study [177].

**Repotrectinib** is an ALK/ROS1/TRK inhibitor [153] and also a potent SRC inhibitor with an IC50 of 5.3 nM. A significant antitumor effect is observed in non-small cell lung cancer patients treated with repotrectinib [178]. Repotrectinib showed significant antitumor effects in neuroblastoma models and was more effective when combined with chemotherapy [179, 180].

### Conclusions and future perspectives

In tumors, FYN is expressed at elevated levels and is involved in many signaling pathways. It phosphorylates downstream signaling proteins, which promotes tumor growth. FYN has been studied in prostate cancer, pancreatic cancer, leukemia, breast cancer, thyroid cancer, bile duct cancer, and other tumors. FYN expression levels of both mRNA and protein were significantly higher in prostate cancer cells than in normal cells. Clinical samples from prostate cancer patients demonstrated that FYN, FAK and PXN expression levels were both increased with significant correlations, hence, FYN might be a prostate cancer molecular target [127]. FYN is downstream of the HGF/MET signaling loop, and HGF can effectively regulate FYN activity, which promotes prostate cancer biology by promoting cell growth and regulating targeted chemotaxis-translocation components in prostate cancer biology [59]. However, it has been demonstrated that the FYN tyrosine kinase gene at chromosome 6q21 is a novel candidate tumor suppressor in prostate cancer and that FYN is downregulated by chromosomal deletion and promoter hypermethylation and expression in prostate cancer [181]. FYN increases prostate cancer cell COX2 activity regardless of changes in COX2 or COX1 protein expression levels. The results of this study depict that FYN phosphorylates human COX2 on Tyr 446 and that the corresponding phosphorylated COX2 activating mutation promotes COX2 activity, and the phosphorylation inactivating mutation prevents the FYN-mediated increase in COX2 activity, which is known to be overexpressed in prostate cancer [182]. Computational analysis of FYN expression in the prostate cancer cell line database demonstrated a correlation between neuroendocrine (NE) markers such as CHGA, CD44, CD56, and SYP expression. FYN contributes to vascular metastasis in progressive prostate cancer [60]. Studies of FYN in prostate cancer have not been entirely consistent, with some studies suggesting that FYN is proliferative and metastatic in prostate cancer, while others suggest that FYN expression is downregulated in prostate cancer and is a tumor suppressor in prostate cancer. However, current studies mainly favor FYN as a proto-oncogene in prostate cancer with oncogenic activity. Second, in breast cancer, detection of FYN levels in clinical samples using immunohistochemical techniques (IHC) revealed that FYN expression levels are significantly higher in breast cancer than in adjacent normal tissues and are an important factor in the poor prognosis of breast cancer [183]. Overexpression of FYN in breast cancer has been reported in the literature, and FYN overexpression promotes cell proliferation, migration, and invasion. In addition, FYN upregulates the expression of mesenchymal markers and epithelial-mesenchymal transition (EMT)-related markers. It downregulates the expression of epithelial markers, and the results suggest that FYN mediates FGF2-induced EMT through FOXO1 transcriptional regulation and via PI3K/AKT and ERK/MAPK pathways [120]. Protein tyrosine phosphatase N23 (PTPN23) is a key player in breast epithelial cells and an inhibitor of cell motility and invasion in breast cancer cells. Knockdown of PTPN23 expression detects tyrosine hyperphosphorylation at the autophosphorylation site in FYN. The overexpression of FYN in breast cancer has been documented, and thus the proliferative phenotype of breast cancer disappears upon inhibition of FYN expression, suggesting that FYN is a downstream molecule of PTPN23 mediating breast carcinogenesis [62]. FYN is required to maintain the basal breast cancer subtype, which is the most aggressive and has mesenchymal features with high metastatic capacity. It has been demonstrated that FYN enhances NOTCH2 activation in basal breast cancer cells through STAT5-mediated upregulation of Jagged-1 and DLL4 NOTCH ligands, thereby contributing to the mesenchymal phenotype. FYN and STAT5 are present at high levels in the positive feedback loop between basal breast cancer cells. FYN directly interacts with STAT5 and increases p-STAT5, which further acts as a transcription factor for FYN [20]. In addition, FYN is associated with tamoxifen resistance in breast cancer, and the above studies establish the role of FYN in promoting tumorigenesis and invasive metastasis in breast cancer. In pancreatic cancer, FYN is associated with tamoxifen resistance. In pancreatic cancer, FYN coordinates with HnRNPA2B1 and Sam68 to regulate apoptosis and promote proliferation and metastasis of pancreatic cancer [184]. Upregulation of FYN expression in pancreatic cancer is associated with pancreatic cancer metastasis, and in pancreatic cancer cells, reduced or absent FYN activity significantly inhibited liver metastasis in nude mice. Active FYN promotes pancreatic cell metastasis by regulating proliferation and apoptosis[128]. It has been demonstrated that Inhibition of FYN activity and/or hnRNP E1 overexpression decreased metastasis in pancreatic cancer cells, and FYN / hnRNP E1 signaling regulated pancreatic cancer metastasis by affecting variable splicing of integrin β1 [97]. Inhibition of FYN expression in pancreatic cancer significantly inhibited proliferation, migration, and invasion of pancreatic cancer cells [29]. In prostate cancer, FYN overexpression, in turn, promotes thyroid cancer cells in colon cancer, and FYN induces early adhesion in colon cancer cells [63]. The next study confirmed that FYN promotes metastatic invasion in colon cancer [122]. In acute lymphoma, FYN interacts with FLT3-ITD to selectively activate STAT5 and induce the transformation of lymphoma cells, and inhibition of FYN may assist in treating patients with acute lymphoma [112]. In addition, FYN expression is associated with acute and chronic leukemia. FYN knockdown or downregulation inhibits the migration and invasion of cholangiocarcinoma cells [123]. However, the relationship between FYN and apoptosis is controversial, and some studies have demonstrated that FYN promotes apoptosis in tumor cells [185]. In advanced neuroblastoma, FYN levels are downregulated and positively correlate with survival in patients with advanced neuroblastoma [186].

However, the current research on the biological role of FYN in tumor inhibition is mainly pro-cancer, but we do not know whether FYN also plays a anti-cancer role in other unstudied tumors. Considering that different tumors have different specificities, studying the role of FYN in other tumors is of great importance. Currently, multiple clinical trials are underway to evaluate inhibitors of FYN/SRC, which not only improve chemotherapy efficacy, [159–161, 174], but also significantly enhance the therapeutic response to immunotherapy and radiotherapy[157, 158]. A variety of preclinical studies have also demonstrated that FYN/SRC inhibitors inhibit multiple tumor progressions[155, 156, 162, 166, 175]. Hence, in order to better understand the activation and inactivation of FYN, as well as the regulation of FYN expression in additional molecules, future studies are needed. The use of FYN inhibitors in patients with high expression or elevated FYN activity will help improve survival rates in cancer patients.

## Data Availability

All data needed to evaluate the conclusions in the paper are present in the paper.

## Abbreviations

TK: Tyrosine Kinases
RTKs: Receptor Protein Tyrosine Kinases
NRPTKs: Non-Receptor Protein Tyrosine Kinases
EGF: Epidermal growth factor
MET: Mesenchymal Epithelial Transition factor
ALK: Anaplastic Lymphoma Kinase
FGF: Fibroblast Growth Factor
VEGF: Vascular Endothelial Growth Factor
DDR: Discoidin Domain Receptor
SFKs: SRC Family of Kinases
SP1: Specificity Protein1
EGR1: Early growth response 1
PRDM14: PR [positive regulatory domain I-binding factor 1 (PRDI-BF1) and retinoblastoma protein-interacting zinc finger gene (RIZ1)] domain containing 14
STAT5: Signal transducers and activators of transcription 5
KLF5: Krüppel-like factor 5
Cbl: Casitas B lineage lymphoma
SHP2: Src homology-2-containing protein tyrosine phosphatase 2
PKA: cAMP-dependent protein kinase A
PTPa: Protein tyrosine phosphatase a
ZAP-70: Zeta-associated protein of 70
PAG: Phosphoprotein associated with glycolipid-enriched membranes
HGF: Hepatocyte growth factor
Wnt: Wingless-related integration site family
Fz2: Frizzled2
Nrf-2: Nuclear factor E2-related factor
LKB1: Live kinase B1
COX2: Cyclooxygenase 2
RSK2: p90 ribosomal S6 kinase 2
G6PD: Glucose-6-phosphate dehydrogenase
ARHGEF16: Rho guanine nucleotide exchange factor 16
PAK-2: p21-activated kinase 2
FAK: Focal adhesion kinase
IBSP: integrin-binding sialoprotein
ADAP: Adhesion and degranulation-promoting adapter protein
SKAP55: Src kinase-associated phosphoprotein of 55 kDa
Nck: Noncatalytic region of tyrosine kinase
RUNX2: Runt-related protein 2
IL-32: Cytokine interleukin-32

## References

[1] Hubbard SR, Till JH (2000). Protein tyrosine kinase structure and function. Annu Rev Biochem.

[2] Scheijen B, Griffin JD (2002). Tyrosine kinase oncogenes in normal hematopoiesis and hematological disease. Oncogene.

[3] Chase A, Cross NC (2006). Signal transduction therapy in haematological malignancies: identification and targeting of tyrosine kinases. Clin Sci (Lond).

[4] Paul MK, Mukhopadhyay AK (2004). Tyrosine kinase - role and significance in Cancer. Int J Med Sci.

[5] Chang YM, Kung HJ, Evans CP (2007). Nonreceptor tyrosine kinases in prostate cancer. Neoplasia.

[6] Semba K, Nishizawa M, Miyajima N, Yoshida MC, Sukegawa J, Yamanashi Y, Sasaki M, Yamamoto T (1986). Toyoshima, yes-related protooncogene, syn, belongs to the protein-tyrosine kinase family. Proc Natl Acad Sci U S A.

[7] Goldsmith JF, Hall CG, Atkinson TP (2002). Identification of an alternatively spliced isoform of the fyn tyrosine kinase. Biochem Biophys Res Commun.

[8] Davidson D, Fournel M, Veillette A (1994). Oncogenic activation of p59fyn tyrosine protein kinase by mutation of its carboxyl-terminal site of tyrosine phosphorylation, tyrosine 528. J Biol Chem.

[9] Johnson LN, Noble ME, Owen DJ (1996). Active and inactive protein kinases: structural basis for regulation. Cell.

[10] Cowan-Jacob SW, Fendrich G, Manley PW, Jahnke W, Fabbro D, Liebetanz J, Meyer T (2005). The crystal structure of a c-Src complex in an active conformation suggests possible steps in c-Src activation. Structure.

[11] Saito YD, Jensen AR, Salgia R (2010). Posadas, Fyn: a novel molecular target in cancer. Cancer.

[12] Appleby MW, Gross JA, Cooke MP, Levin SD, Qian X, Perlmutter RM (1992). Defective T cell receptor signaling in mice lacking the thymic isoform of p59fyn. Cell.

[13] Belsches-Jablonski AP, Biscardi JS, Peavy DR, Tice DA, Romney DA, Parsons SJ (2001). Src family kinases and HER2 interactions in human breast cancer cell growth and survival. Oncogene.

[14] Calautti E, Grossi M, Mammucari C, Aoyama Y, Pirro M, Ono Y, Li J, Dotto GP (2002). Fyn tyrosine kinase is a downstream mediator of Rho/PRK2 function in keratinocyte cell-cell adhesion. J Cell Biol.

[15] Cary LA, Chang JF, Guan JL (1996). Stimulation of cell migration by overexpression of focal adhesion kinase and its association with src and fyn. J Cell Sci.

[16] Kinsey WH (2014). SRC-family tyrosine kinases in oogenesis, oocyte maturation and fertilization: an evolutionary perspective. Adv Exp Med Biol.

[17] Sugie K, Kawakami T, Maeda Y, Kawabe T, Uchida A, Yodoi J (1991). Fyn tyrosine kinase associated with fc epsilon RII/CD23: possible multiple roles in lymphocyte activation. Proc Natl Acad Sci U S A.

[18] Gao Y, Howard A, Ban K, Chandra J (2009). Oxidative stress promotes transcriptional up-regulation of Fyn in BCR-ABL1-expressing cells. J Biol Chem.

[19] Moriya C, Taniguchi H, Miyata K, Nishiyama N, Kataoka K, Imai K (2017). Inhibition of PRDM14 expression in pancreatic cancer suppresses cancer stem-like properties and liver metastasis in mice. Carcinogenesis.

[20] Lee GH, Yoo KC, An Y, Lee HJ, Lee M, Uddin N, Kim MJ, Kim IG, Suh Y, Lee SJ (2018). FYN promotes mesenchymal phenotypes of basal type breast cancer cells through STAT5/NOTCH2 signaling node. Oncogene.

[21] Juric D, Lacayo NJ, Ramsey MC, Racevskis J, Wiernik PH, Rowe JM, Goldstone AH, O’Dwyer PJ, Paietta E, Sikic BI (2007). Differential gene expression patterns and interaction networks in BCR-ABL-positive and -negative adult acute lymphoblastic leukemias. J Clin Oncol.

[22] Du C, Gao Y, Xu S, Jia J, Huang Z, Fan J, Wang X, He D, Guo P (2016). KLF5 promotes cell migration by up-regulating FYN in bladder cancer cells. FEBS Lett.

[23] Ninio-Many L, Grossman H, Shomron N, Chuderland D, Shalgi R (2013). microRNA-125a-3p reduces cell proliferation and migration by targeting fyn. J Cell Sci.

[24] Grossman H, Chuderland D, Ninio-Many L, Hasky N, Kaplan-Kraicer R, Shalgi R (2015). A novel regulatory pathway in granulosa cells, the LH/human chorionic gonadotropin-microRNA-125a-3p-Fyn pathway, is required for ovulation. FASEB J.

[25] Wang J, Zheng Y, Bai B, Song Y, Zheng K, Xiao J, Liang Y, Bao L, Zhou Q, Ji L, Feng X (2020). MicroRNA-125a-3p participates in odontoblastic differentiation of dental pulp stem cells by targeting fyn. Cytotechnology.

[26] Liu G, Ji L, Ke M, Ou Z, Tang N, Li Y (2018). miR-125a-3p is responsible for chemosensitivity in PDAC by inhibiting epithelial-mesenchymal transition via Fyn. Biomed Pharmacother.

[27] Ninio-Many L, Grossman H, Levi M, Zilber S, Tsarfaty I, Shomron N, Tuvar A, Chuderland D, Stemmer SM, Ben-Aharon I, Shalgi R (2014). MicroRNA miR-125a-3p modulates molecular pathway of motility and migration in prostate cancer cells. Oncoscience.

[28] Xu Q, Liu Y, Pan H, Xu T, Li Y, Yuan J, Li P, Yao W, Yan W, Ni C (2019). Aberrant expression of miR-125a-3p promotes fibroblast activation via Fyn/STAT3 pathway during silica-induced pulmonary fibrosis. Toxicology.

[29] Liu D, Gao M, Wu K, Zhu D, Yang Y, Zhao S (2019). LINC00152 facilitates tumorigenesis in esophageal squamous cell carcinoma via miR-153-3p/FYN axis. Biomed Pharmacother.

[30] Mi H, Wang X, Wang F, Li L, Zhu M, Wang N, Xiong Y, Gu Y (2018). miR-381 induces sensitivity of breast cancer cells to doxorubicin by inactivation of MAPK signaling via FYN. Eur J Pharmacol.

[31] Yao X, Xian X, Fang M, Fan S, Li W (2020). Loss of miR-369 promotes tau phosphorylation by targeting the fyn and Serine/Threonine-Protein kinase 2 signaling pathways in Alzheimer’s Disease mice. Front Aging Neurosci.

[32] Basak I, Bhatlekar S, Manne BK, Stoller M, Hugo S, Kong X, Ma L, Rondina MT, Weyrich AS, Edelstein LC, Bray PF (2019). miR-15a-5p regulates expression of multiple proteins in the megakaryocyte GPVI signaling pathway. J Thromb Haemost.

[33] Ambrozkiewicz MC, Schwark M, Kishimoto-Suga M, Borisova E, Hori K, Salazar-Lazaro A, Rusanova A, Altas B, Piepkorn L, Bessa P, Schaub T, Zhang X, Rabe T, Ripamonti S, Rosario M, Akiyama H, Jahn O, Kobayashi T, Hoshino M, Tarabykin V, Kawabe H (2018). Polarity acquisition in cortical neurons is driven by synergistic action of Sox9-regulated Wwp1 and Wwp2 E3 ubiquitin ligases and intronic miR-140. Neuron..

[34] Meng Y, Quan L, Liu A (2018). Identification of key microRNAs associated with diffuse large B-cell lymphoma by analyzing serum microRNA expressions. Gene.

[35] Sriroopreddy R, Sajeed R, P R (2019). Differentially expressed gene (DEG) based protein-protein interaction (PPI) network identifies a spectrum of gene interactome, transcriptome and correlated miRNA in nondisjunction down syndrome. Int J Biol Macromol.

[36] Colden M, Dar AA, Saini S, Dahiya PV, Shahryari V, Yamamura S, Tanaka Y, Stein G, Dahiya R, Majid S (2017). MicroRNA-466 inhibits tumor growth and bone metastasis in prostate cancer by direct regulation of osteogenic transcription factor RUNX2. Cell Death Dis.

[37] Joshi T, Elias D, Stenvang J, Alves CL, Teng F, Lyng MB, Lykkesfeldt AE, Brunner N, Wang J, Gupta R, Workman CT, Ditzel HJ (2016). Integrative analysis of miRNA and gene expression reveals regulatory networks in tamoxifen-resistant breast cancer. Oncotarget.

[38] Seok HJ, Choi YE, Choi JY, Yi JM, Kim EJ, Choi MY, Lee SJ, Bae IH (2021). Novel mir-5088-5p promotes malignancy of breast cancer by inhibiting DBC2. Mol Ther Nucleic Acids.

[39] Rao N, Ghosh AK, Douillard P, Andoniou CE, Zhou P, Band H (2002). An essential role of ubiquitination in Cbl-mediated negative regulation of the src-family kinase fyn. Signal Transduct.

[40] Andoniou CE, Lill NL, Thien CB, Lupher ML, Ota S, Bowtell DD, Scaife RM, Langdon WY, Band H (2000). The Cbl proto-oncogene product negatively regulates the src-family tyrosine kinase fyn by enhancing its degradation. Mol Cell Biol.

[41] Li X, Yang Y, Hu Y, Dang D, Regezi J, Schmidt BL, Atakilit A, Chen B, Ellis D, Ramos DM (2003). Alphavbeta6-Fyn signaling promotes oral cancer progression. J Biol Chem.

[42] Yang X, Dutta U, Shaw LM (2010). SHP2 mediates the localized activation of Fyn downstream of the alpha6beta4 integrin to promote carcinoma invasion. Mol Cell Biol.

[43] Mukherjee A, Singh R, Udayan S, Biswas S, Reddy PP, Manmadhan S, George G, Kumar S, Das R, Rao BM, Gulyani A (2020). A fyn biosensor reveals pulsatile, spatially localized kinase activity and signaling crosstalk in live mammalian cells. Elife.

[44] Lu KV, Zhu S, Cvrljevic A, Huang TT, Sarkaria S, Ahkavan D, Dang J, Dinca EB, Plaisier SB, Oderberg I, Lee Y, Chen Z, Caldwell JS, Xie Y, Loo JA, Seligson D, Chakravari A, Lee FY, Weinmann R, Cloughesy TF, Nelson SF, Bergers G, Graeber T, Furnari FB, James CD, Cavenee WK, Johns TG, Mischel PS (2009). Fyn and SRC are effectors of oncogenic epidermal growth factor receptor signaling in glioblastoma patients. Cancer Res.

[45] Yadav V, Denning MF (2011). Fyn is induced by Ras/PI3K/Akt signaling and is required for enhanced invasion/migration. Mol Carcinog.

[46] Hansen K, Alonso G, Courtneidge SA, Ronnstrand L, Heldin CH (1997). PDGF-induced phosphorylation of Tyr28 in the N-terminus of Fyn affects fyn activation. Biochem Biophys Res Commun.

[47] Yeo MG, Oh HJ, Cho HS, Chun JS, Marcantonio EE, Song WK (2011). Phosphorylation of ser 21 in Fyn regulates its kinase activity, focal adhesion targeting, and is required for cell migration. J Cell Physiol.

[48] Konig S, Nimtz M, Scheiter M, Ljunggren HG, Bryceson YT, Jansch L (2012). Kinome analysis of receptor-induced phosphorylation in human natural killer cells. PLoS ONE.

[49] Pires de Miranda M, Alenquer M, Marques S, Rodrigues L, Lopes F, Bustelo XR, Simas JP (2008). The Gammaherpesvirus m2 protein manipulates the Fyn/Vav pathway through a multidocking mechanism of assembly. PLoS ONE.

[50] Rodrigues L, Pires de Miranda M, Caloca MJ, Bustelo XR, Simas JP (2006). Activation of Vav by the gammaherpesvirus M2 protein contributes to the establishment of viral latency in B lymphocytes. J Virol.

[51] Maksumova L, Le HT, Muratkhodjaev F, Davidson D, Veillette A, Pallen CJ (2005). Protein tyrosine phosphatase alpha regulates fyn activity and Cbp/PAG phosphorylation in thymocyte lipid rafts. J Immunol.

[52] Bamberger M, Santos AM, Goncalves CM, Oliveira MI, James JR, Moreira A, Lozano F, Davis SJ, Carmo AM (2011). A new pathway of CD5 glycoprotein-mediated T cell inhibition dependent on inhibitory phosphorylation of fyn kinase. J Biol Chem.

[53] Piedra J, Miravet S, Castano J, Palmer HG, Heisterkamp N, Garcia de Herreros A, Dunach M (2003). p120 catenin-associated fer and fyn tyrosine kinases regulate beta-catenin Tyr-142 phosphorylation and beta-catenin-alpha-catenin Interaction. Mol Cell Biol.

[54] McPherson VA, Sharma N, Everingham S, Smith J, Zhu HH, Feng GS, Craig AW (2009). SH2 domain-containing phosphatase-2 protein-tyrosine phosphatase promotes fc epsilon RI-induced activation of Fyn and Erk pathways leading to TNF alpha release from bone marrow-derived mast cells. J Immunol.

[55] Choi KY, Napper S, Mookherjee N (2014). Human cathelicidin LL-37 and its derivative IG-19 regulate interleukin-32-induced inflammation. Immunology.

[56] Yasuda K, Nagafuku M, Shima T, Okada M, Yagi T, Yamada T, Minaki Y, Kato A, Tani-Ichi S, Hamaoka T, Kosugi A (2002). Cutting edge: Fyn is essential for tyrosine phosphorylation of Csk-binding protein/phosphoprotein associated with glycolipid-enriched microdomains in lipid rafts in resting T cells. J Immunol.

[57] Solheim SA, Torgersen KM, Tasken K, Berge T (2008). Regulation of FynT function by dual domain docking on PAG/Cbp. J Biol Chem.

[58] Huang J, Asawa T, Takato T, Sakai R (2003). Cooperative roles of Fyn and cortactin in cell migration of metastatic murine melanoma. J Biol Chem.

[59] Jensen AR, David SY, Liao C, Dai J, Keller ET, Al-Ahmadie H, Dakin-Hache K, Usatyuk P, Sievert MF, Paner GP, Yala S, Cervantes GM, Natarajan V, Salgia R, Posadas EM (2011). Fyn is downstream of the HGF/MET signaling axis and affects cellular shape and tropism in PC3 cells. Clin Cancer Res.

[60] Gururajan M, Cavassani KA, Sievert M, Duan P, Lichterman J, Huang JM, Smith B, You S, Nandana S, Chu GC, Mink S, Josson S, Liu C, Morello M, Jones LW, Kim J, Freeman MR, Bhowmick N, Zhau HE, Chung LW (2015). Posadas, SRC family kinase FYN promotes the neuroendocrine phenotype and visceral metastasis in advanced prostate cancer. Oncotarget.

[61] Villarroel A, Valle-Perez BD, Fuertes G, Curto J, Ontiveros N, Garcia de Herreros A, Dunach M (2020). Src and fyn define a new signaling cascade activated by canonical and non-canonical wnt ligands and required for gene transcription and cell invasion. Cell Mol Life Sci.

[62] Zhang S, Fan G, Hao Y, Hammell M, Wilkinson JE, Tonks NK (2017). Suppression of protein tyrosine phosphatase N23 predisposes to breast tumorigenesis via activation of FYN kinase. Genes Dev.

[63] Baillat G, Siret C, Delamarre E, Luis J (2008). Early adhesion induces interaction of FAK and Fyn in lipid domains and activates raft-dependent akt signaling in SW480 colon cancer cells. Biochim Biophys Acta.

[64] Ha JH, Jayaraman M, Yan M, Dhanasekaran P, Isidoro C, Song YS, Dhanasekaran DN (2021). GNAi2/gip2-Regulated transcriptome and its therapeutic significance in Ovarian Cancer. Biomolecules.

[65] Abouzaglou J, Benistant C, Gimona M, Roustan C, Kassab R, Fattoum A (2004). Tyrosine phosphorylation of calponins. Inhibition of the interaction with F-actin. Eur J Biochem.

[66] Yamada E, Okada S, Bastie CC, Vatish M, Nakajima Y, Shibusawa R, Ozawa A, Pessin JE, Yamada M (2016). Fyn phosphorylates AMPK to inhibit AMPK activity and AMP-dependent activation of autophagy. Oncotarget.

[67] Wallace TA, Xia SL, Sayeski PP (2005). Jak2 tyrosine kinase prevents angiotensin II-mediated inositol 1,4,5 trisphosphate receptor degradation. Vascul Pharmacol.

[68] Gotoh H, Okumura N, Yagi T, Okumura A, Shima T, Nagai K (2006). Fyn-induced phosphorylation of beta-adducin at tyrosine 489 and its role in their subcellular localization. Biochem Biophys Res Commun.

[69] Paronetto MP, Achsel T, Massiello A, Chalfant CE, Sette C (2007). The RNA-binding protein Sam68 modulates the alternative splicing of Bcl-x. J Cell Biol.

[70] Lim SH, Kwon SK, Lee MK, Moon J, Jeong DG, Park E, Kim SJ, Park BC, Lee SC, Ryu SE, Yu DY, Chung BH, Kim E, Myung PK, Lee JR (2009). Synapse formation regulated by protein tyrosine phosphatase receptor T through interaction with cell adhesion molecules and fyn. EMBO J.

[71] Kaspar JW, Jaiswal AK (2011). Tyrosine phosphorylation controls nuclear export of Fyn, allowing Nrf2 activation of cytoprotective gene expression. FASEB J.

[72] Qian D, Lev S, van Oers NS, Dikic I, Schlessinger J, Weiss A (1997). Tyrosine phosphorylation of Pyk2 is selectively regulated by fyn during TCR signaling. J Exp Med.

[73] Yamada E, Pessin JE, Kurland IJ, Schwartz GJ, Bastie CC (2010). Fyn-dependent regulation of energy expenditure and body weight is mediated by tyrosine phosphorylation of LKB1. Cell Metab.

[74] Hatton O, Lambert SL, Krams SM, Martinez OM (2012). Src kinase and syk activation initiate PI3K signaling by a chimeric latent membrane protein 1 in Epstein-Barr virus (EBV) + B cell lymphomas. PLoS ONE.

[75] Ghosh AK, Reddi AL, Rao NL, Duan L, Band V, Band H (2004). Biochemical basis for the requirement of kinase activity for Cbl-dependent ubiquitinylation and degradation of a target tyrosine kinase. J Biol Chem.

[76] Jacobson M, Redfern RE, Mills GD (1975). Naturally occurring insect growth regulators. III. Echinolone, a highly active juvenile hormone mimic from Echinacea angustifolia roots. Lloydia.

[77] Boivin D, Labbe D, Fontaine N, Lamy S, Beaulieu E, Gingras D, Beliveau R (2009). The stem cell marker CD133 (prominin-1) is phosphorylated on cytoplasmic tyrosine-828 and tyrosine-852 by src and fyn tyrosine kinases. Biochemistry.

[78] Zhang J, Li D, Yue X, Zhang M, Liu P, Li G (2018). Colorimetric in situ assay of membrane-bound enzyme based on lipid bilayer inhibition of ion transport. Theranostics.

[79] He Z, Cho YY, Ma WY, Choi HS, Bode AM, Dong Z (2005). Regulation of ultraviolet B-induced phosphorylation of histone H3 at serine 10 by fyn kinase. J Biol Chem.

[80] Cui J, Matkovich SJ, deSouza N, Li S, Rosemblit N, Marks AR (2004). Regulation of the type 1 inositol 1,4,5-trisphosphate receptor by phosphorylation at tyrosine 353. J Biol Chem.

[81] Chesarino NM, McMichael TM, Hach JC, Yount JS (2014). Phosphorylation of the antiviral protein interferon-inducible transmembrane protein 3 (IFITM3) dually regulates its endocytosis and ubiquitination. J Biol Chem.

[82] Yamada E, Bastie CC (2014). Disruption of Fyn SH3 domain interaction with a proline-rich motif in liver kinase B1 results in activation of AMP-activated protein kinase. PLoS ONE.

[83] Samovski D, Sun J, Pietka T, Gross RW, Eckel RH, Su X, Stahl PD, Abumrad NA (2015). Regulation of AMPK activation by CD36 links fatty acid uptake to beta-oxidation. Diabetes.

[84] Niture SK, Khatri R, Jaiswal AK (2014). Regulation of Nrf2-an update. Free Radic Biol Med.

[85] Jain AK, Jaiswal AK (2006). Phosphorylation of tyrosine 568 controls nuclear export of Nrf2. J Biol Chem.

[86] Chamorro M, Czar MJ, Debnath J, Cheng G, Lenardo MJ, Varmus HE, Schwartzberg PL (2001). Requirements for activation and RAFT localization of the T-lymphocyte kinase Rlk/Txk. BMC Immunol.

[87] Kang S, Dong S, Guo A, Ruan H, Lonial S, Khoury HJ, Gu TL, Chen J (2008). Epidermal growth factor stimulates RSK2 activation through activation of the MEK/ERK pathway and src-dependent tyrosine phosphorylation of RSK2 at Tyr-529. J Biol Chem.

[88] Feuillet V, Semichon M, Restouin A, Harriague J, Janzen J, Magee A, Collette Y, Bismuth G (2002). The distinct capacity of Fyn and Lck to phosphorylate Sam68 in T cells is essentially governed by SH3/SH2-catalytic domain linker interactions. Oncogene.

[89] Fusaki N, Matsuda S, Nishizumi H, Umemori H, Yamamoto T (1996). Physical and functional interactions of protein tyrosine kinases, p59fyn and ZAP-70, in T cell signaling. J Immunol.

[90] Sun M, Sheng H, Wu T, Song J, Sun H, Wang Y, Wang J, Li Z, Zhao H, Tan J, Li Y, Chen G, Huang Q, Zhang Y, Lan B, Liu S, Shan C, Zhang S (2021). PIKE-A promotes glioblastoma growth by driving PPP flux through increasing G6PD expression mediated by phosphorylation of STAT3. Biochem Pharmacol.

[91] Zhang X, Huang Z, Guo Y, Xiao T, Tang L, Zhao S, Wu L, Su J, Zeng W, Huang H, Li Z, Tao J, Zhou J, Chen X, Peng C (2020). The phosphorylation of CD147 by Fyn plays a critical role for melanoma cells growth and metastasis. Oncogene.

[92] Zhang X, Huang Z, Guo Y, Xiao T, Tang L, Zhao S, Wu L, Su J, Zeng W, Huang H, Li Z, Tao J, Zhou J, Chen X, Peng C (2020). Correction to: the phosphorylation of CD147 by Fyn plays a critical role for melanoma cells growth and metastasis. Oncogene.

[93] Yu B, Xu L, Chen L, Wang Y, Jiang H, Wang Y, Yan Y, Luo S, Zhai Z (2020). FYN is required for ARHGEF16 to promote proliferation and migration in colon cancer cells. Cell Death Dis.

[94] Du G, Wang J, Zhang T, Ding Q, Jia X, Zhao X, Dong J, Yang X, Lu S, Zhang C, Liu Z, Zeng Z, Safadi R, Qi R, Zhao X, Hong Z, Lu Y (2020). Targeting src family kinase member fyn by Saracatinib attenuated liver fibrosis in vitro and in vivo. Cell Death Dis.

[95] Elias D, Vever H, Laenkholm AV, Gjerstorff MF, Yde CW, Lykkesfeldt AE, Ditzel HJ (2015). Gene expression profiling identifies FYN as an important molecule in tamoxifen resistance and a predictor of early recurrence in patients treated with endocrine therapy. Oncogene.

[96] Liu R, Li W, Tao B, Wang X, Yang Z, Zhang Y, Wang C, Liu R, Gao H, Liang J, Yang W (2019). Tyrosine phosphorylation activates 6-phosphogluconate dehydrogenase and promotes tumor growth and radiation resistance. Nat Commun.

[97] Jiang P, Li Z, Tian F, Li X, Yang J (2017). Fyn/heterogeneous nuclear ribonucleoprotein E1 signaling regulates pancreatic cancer metastasis by affecting the alternative splicing of integrin beta1. Int J Oncol.

[98] Yang J, Wang Y, Zeng Z, Qiao L, Zhuang L, Gao Q, Ma D, Huang X (2017). Smad4 deletion in blood vessel endothelial cells promotes ovarian cancer metastasis. Int J Oncol.

[99] Dong W, Sun SJ, Qin JJ, Liu GM (2020). Fyn stimulates the progression of pancreatic cancer via Fyn-GluN2b-AKT axis. Eur Rev Med Pharmacol Sci.

[100] Moon CS, Reglero C, Cortes JR, Quinn SA, Alvarez S, Zhao J, Lin WW, Cooke AJ, Abate F, Soderquist CR, Finana C, Inghirami G, Campo E, Bhagat G, Rabadan R, Palomero T, Ferrando AA (2021). FYN-TRAF3IP2 induces NF-kappaB signaling-driven peripheral T cell lymphoma. Nat Cancer.

[101] Tominaga T, Sahai E, Chardin P, McCormick F, Courtneidge SA, Alberts AS (2000). Diaphanous-related formins bridge rho GTPase and src tyrosine kinase signaling. Mol Cell.

[102] Ng MM, Chang F, Burgess DR (2005). Movement of membrane domains and requirement of membrane signaling molecules for cytokinesis. Dev Cell.

[103] Okamoto M, Nakayama Y, Kakihana A, Yuki R, Yamaguchi N, Yamaguchi N (2016). Fyn accelerates M phase progression by promoting the Assembly of Mitotic Spindle Microtubules. J Cell Biochem.

[104] Levi M, Maro B, Shalgi R (2010). Fyn kinase is involved in cleavage furrow ingression during meiosis and mitosis. Reproduction.

[105] Czech DA, Vander JM, Zanden (1991). Drinking behavior in the spiny mouse (Acomys cahirinus) following putative dipsogenic challenges. Pharmacol Biochem Behav.

[106] Fenton SE, Hutchens KA, Denning MF (2015). Targeting fyn in ras-transformed cells induces F-actin to promote adherens junction-mediated cell-cell adhesion. Mol Carcinog.

[107] Chapman NM, Yoder AN, Houtman JC (2012). Non-catalytic functions of Pyk2 and fyn regulate late stage adhesion in human T cells. PLoS ONE.

[108] Gang EJ, Kim HN, Hsieh YT, Ruan Y, Ogana HA, Lee S, Pham J, Geng H, Park E, Klemm L, Willman CL, Carroll WL, Mittelman SD, Orgel E, Oberley MJ, Parekh C, Abdel-Azim H, Bhojwani D, Wayne AS, De Arcangelis A, Georges-Labouesse E, Wayner E, Bonig H, Minasyan A, Hoeve JT, Graeber TG, Muschen M, Heisterkamp N, Kim YM (2020). Integrin alpha6 mediates the drug resistance of acute lymphoblastic B-cell leukemia. Blood.

[109] Zheng J, Li H, Xu D, Zhu H (2017). Upregulation of tyrosine kinase FYN in human thyroid carcinoma: role in modulating Tumor Cell Proliferation, Invasion, and Migration. Cancer Biother Radiopharm.

[110] Singh MM, Howard A, Irwin ME, Gao Y, Lu X, Multani A, Chandra J (2012). Expression and activity of Fyn mediate proliferation and blastic features of chronic myelogenous leukemia. PLoS ONE.

[111] Kim HJ, Warren JT, Kim SY, Chappel JC, DeSelm CJ, Ross FP, Zou W, Teitelbaum SL (2010). Fyn promotes proliferation, differentiation, survival and function of osteoclast lineage cells. J Cell Biochem.

[112] Chougule RA, Kazi JU, Ronnstrand L (2016). FYN expression potentiates FLT3-ITD induced STAT5 signaling in acute myeloid leukemia. Oncotarget.

[113] Zhang S, Qi Q, Chan CB, Zhou W, Chen J, Luo HR, Appin C, Brat DJ, Ye K (2016). Fyn-phosphorylated PIKE-A binds and inhibits AMPK signaling, blocking its tumor suppressive activity. Cell Death Differ.

[114] Je DW, O YM, Ji YG, Cho Y, Lee DH (2014). The inhibition of SRC family kinase suppresses pancreatic cancer cell proliferation, migration, and invasion. Pancreas..

[115] Zhao L, Li W, Marshall C, Griffin T, Hanson M, Hick R, Dentchev T, Williams E, Werth A, Miller C, Bashir H, Pear W, Seykora JT (2009). Srcasm inhibits Fyn-induced cutaneous carcinogenesis with modulation of Notch1 and p53. Cancer Res.

[116] Tang L, Long J, Li K, Zhang X, Chen X, Peng C (2020). A novel chalcone derivative suppresses melanoma cell growth through targeting Fyn/Stat3 pathway. Cancer Cell Int.

[117] Guo S, Ran H, Xiao D, Huang H, Mi L, Wang X, Chen L, Li D, Zhang S, Han Q, Zhou T, Li A, Man J (2019). NT5DC2 promotes tumorigenicity of glioma stem-like cells by upregulating fyn. Cancer Lett.

[118] Ansieau S (2013). EMT in breast cancer stem cell generation. Cancer Lett.

[119] Sarkar FH, Li Y, Wang Z, Kong D (2009). Pancreatic cancer stem cells and EMT in drug resistance and metastasis. Minerva Chir.

[120] Xie YG, Yu Y, Hou LK, Wang X, Zhang B, Cao XC (2016). FYN promotes breast cancer progression through epithelial-mesenchymal transition. Oncol Rep.

[121] Lewin B, Siu A, Baker C, Dang D, Schnitt R, Eisapooran P, Ramos DM (2010). Expression of fyn kinase modulates EMT in oral cancer cells. Anticancer Res.

[122] Gujral TS, Chan M, Peshkin L, Sorger PK, Kirschner MW, MacBeath G (2014). A noncanonical Frizzled2 pathway regulates epithelial-mesenchymal transition and metastasis. Cell.

[123] Lyu SC, Han DD, Li XL, Ma J, Wu Q, Dong HM, Bai C, He Q (2018). Fyn knockdown inhibits migration and invasion in cholangiocarcinoma through the activated AMPK/mTOR signaling pathway. Oncol Lett.

[124] Schneble N, Muller J, Kliche S, Bauer R, Wetzker R, Bohmer FD, Wang ZQ, Muller JP (2017). The protein-tyrosine phosphatase DEP-1 promotes migration and phagocytic activity of microglial cells in part through negative regulation of fyn tyrosine kinase. Glia.

[125] Lamalice L, Houle F, Huot J (2006). Phosphorylation of Tyr1214 within VEGFR-2 triggers the recruitment of Nck and activation of Fyn leading to SAPK2/p38 activation and endothelial cell migration in response to VEGF. J Biol Chem.

[126] Bernardini G, Kim JY, Gismondi A, Butcher EC, Santoni A (2005). Chemoattractant induces LFA-1 associated PI 3K activity and cell migration that are dependent on fyn signaling. FASEB J.

[127] Posadas EM, Al-Ahmadie H, Robinson VL, Jagadeeswaran R, Otto K, Kasza KE, Tretiakov M, Siddiqui J, Pienta KJ, Stadler WM, Rinker-Schaeffer C, Salgia R (2009). FYN is overexpressed in human prostate cancer. BJU Int.

[128] Chen ZY, Cai L, Bie P, Wang SG, Jiang Y, Dong JH, Li XW (2010). Roles of Fyn in pancreatic cancer metastasis. J Gastroenterol Hepatol.

[129] Huang C, Huang YL, Wang CC, Pan YL, Lai YH, Huang HC (2019). Ampelopsins A and C induce apoptosis and metastasis through Downregulating AxL, TYRO3, and FYN Expressions in MDA-MB-231 breast Cancer cells. J Agric Food Chem.

[130] Chen Y, Qin Y, Dai M, Liu L, Ni Y, Sun Q, Li L, Zhou Y, Qiu C, Jiang Y (2021). IBSP, a potential recurrence biomarker, promotes the progression of colorectal cancer via Fyn/beta-catenin signaling pathway. Cancer Med.

[131] Yu J, Zhou Z, Wei Z, Wu J, OuYang J, Huang W, He Y, Zhang C (2020). FYN promotes gastric cancer metastasis by activating STAT3-mediated epithelial-mesenchymal transition. Transl Oncol.

[132] Jiang C, Liu Y, Wen S, Xu C, Gu L (2021). In silico development and clinical validation of novel 8 gene signature based on lipid metabolism related genes in colon adenocarcinoma. Pharmacol Res.

[133] Sheng Sun Y, Liu M, Zhou J, Wen (2022). PA2G4 promotes the metastasis of hepatocellular carcinoma by stabilizing FYN mRNA in a YTHDF2-dependent manner. Cell & Bioscience.

[134] Irwin ME, Johnson BP, Manshouri R, Amin HM, Chandra J (2015). A NOX2/Egr-1/Fyn pathway delineates new targets for TKI-resistant malignancies. Oncotarget.

[135] Fenouille N, Puissant A, Dufies M, Robert G, Jacquel A, Ohanna M, Deckert M, Pasquet JM, Mahon FX, Cassuto JP, Raynaud S, Tartare-Deckert S, Auberger P (2010). Persistent activation of the Fyn/ERK kinase signaling axis mediates imatinib resistance in chronic myelogenous leukemia cells through upregulation of intracellular SPARC. Cancer Res.

[136] Airiau K, Turcq B, Mahon FX, Belloc F (2017). A new mechanism of resistance to ABL1 tyrosine kinase inhibitors in a BCR-ABL1-positive cell line. Leuk Res.

[137] Grosso S, Puissant A, Dufies M, Colosetti P, Jacquel A, Lebrigand K, Barbry P, Deckert M, Cassuto JP, Mari B, Auberger P (2009). Gene expression profiling of imatinib and PD166326-resistant CML cell lines identifies Fyn as a gene associated with resistance to BCR-ABL inhibitors. Mol Cancer Ther.

[138] Ergün S, Akgün O, Hekim NT, Aslan S, Ari F (2022). The interrelationship between fyn and Mir-128/193a-5p/494 in imatinib resistance in prostate cancer. Anti-Cancer Agents Med Chem.

[139] Abram CL, Lowell CA (2008). The diverse functions of src family kinases in macrophages. Front Biosci.

[140] Sugie K, Jeon MS, Grey HM (2004). Activation of naive CD4 T cells by anti-CD3 reveals an important role for Fyn in Lck-mediated signaling. Proc Natl Acad Sci U S A.

[141] Sillaber C, Herrmann H, Bennett K, Rix U, Baumgartner C, Bohm A, Herndlhofer S, Tschachler E, Superti-Furga G, Jager U, Valent P (2009). Immunosuppression and atypical infections in CML patients treated with dasatinib at 140 mg daily. Eur J Clin Invest.

[142] Wu N, Song H, Veillette A (2021). Plasma membrane lipid scrambling causing phosphatidylserine exposure negatively regulates NK cell activation. Cell Mol Immunol.

[143] Malarkannan S (2020). Molecular mechanisms of FasL-mediated ‘reverse-signaling’. Mol Immunol.

[144] Comba A, Dunn PJ, Argento AE, Kadiyala P, Ventosa M, Patel P, Zamler DB, Nunez FJ, Zhao L, Castro MG, Lowenstein PR (2020). Fyn tyrosine kinase, a downstream target of receptor tyrosine kinases, modulates antiglioma immune responses. Neuro Oncol.

[145] Gerbec ZJ, Thakar MS, Malarkannan S (2015). The Fyn-ADAP Axis: cytotoxicity Versus Cytokine production in Killer cells. Front Immunol.

[146] Li C, Li W, Xiao J, Jiao S, Teng F, Xue S, Zhang C, Sheng C, Leng Q, Rudd CE, Wei B, Wang H (2015). ADAP and SKAP55 deficiency suppresses PD-1 expression in CD8 + cytotoxic T lymphocytes for enhanced anti-tumor immunotherapy. EMBO Mol Med.

[147] Edwin M, Posadas RS, Ahmed T, Karrison, Russell Z, Szmulewitz (2016). Saracatinib as a metastasis inhibitor in metastatic castration-resistant prostate cancer: a University of Chicago Phase 2 Consortium and DOD/PCF prostate Cancer clinical trials Consortium Study. Prostate.

[148] Harriet M, Kluger MD, Arkadiuz Z, Dudek MD, Carrie McCann RN (2017). A phase 2 trial of dasatinib in advanced melanoma. Cancer..

[149] I R H M Konings MJA, de Jonge H, Burger A, van der Gaast LEC, van Beijsterveldt (2010). Phase I and pharmacological study of the broad-spectrum tyrosine kinase inhibitor JNJ-26483327 in patients with advanced solid tumours. Br J Cancer.

[150] Woodcock VK, Clive S, Wilson RH, Coyle VM, Stratford MRL (2018). A first-in-human phase I study to determine the maximum tolerated dose of the oral Src/ABL inhibitor AZD0424. Br J Cancer.

[151] Molina JR, Foster NR, Reungwetwattana T, Nelson GD (2014). A phase II trial of the src-kinase inhibitor saracatinib after four cycles of chemotherapy for patients with extensive stage small cell lung cancer. Lung Cancer.

[152] Scher HI, Halabi S, Tannock I, Morris M, Sternberg CN, Prostate Cancer Clinical Trials Working Group (2008). Design and end points of clinical trials for patients with progressive prostate cancer and castrate levels of testosterone: recommendations of the prostate Cancer clinical trials Working Group. J Clin Oncol.

[153] Drilon A, Ou SI, Cho BC, Kim DW (2018). Repotrectinib (TPX-0005) is a next-generation ROS1/TRK/ALK inhibitor that potently inhibits ROS1/TRK/ALK solvent- front mutations. Cancer Discov.

[154] Ammer AG, Kelley LC, Hayes KE, Evans JV (2009). Saracatinib impairs Head and Neck squamous cell Carcinoma Invasion by disrupting Invadopodia function. J Cancer Sci Ther.

[155] Nam HJ, Im SA, Oh DY, Elvin P, Kim HP, Yoon YK, Min A, Song SH, Han SW, Kim TY, Bang YJ (2013). Antitumor activity of saracatinib (AZD0530), a c-Src/Abl kinase inhibitor, alone or in combination with chemotherapeutic agents in gastric cancer. Mol Cancer Ther.

[156] Cavalloni G, Peraldo-Neia C, Sarotto I, Gammaitoni L (2012). Antitumor activity of src inhibitor saracatinib (AZD-0530) in preclinical models of biliary tract carcinomas. Mol Cancer Ther.

[157] Yun HS, Lee J, Kil WJ, Kramp TR, Tofilon PJ, Camphausen K (2021). The Radiosensitizing Effect of AZD0530 in Glioblastoma and Glioblastoma Stem-Like cells. Mol Cancer Ther.

[158] Kadota H, Yuge R, Shimizu D, Miyamoto R, Otani R, Hiyama Y, Takigawa H, Hayashi R, Urabe Y, Kitadai Y, Oka S, Tanaka S (2022). Anti-programmed cell Death-1 antibody and Dasatinib Combination Therapy Exhibits Efficacy in Metastatic Colorectal Cancer Mouse Models. Cancers (Basel).

[159] Wang H, Lu Y, Wang M, Shen A, Wu Y, Xu X, Li Y (2022). Src inhibitor dasatinib sensitized gastric cancer cells to cisplatin. Med Oncol.

[160] Xiao J, Xu M, Hou T, Huang Y, Yang C, Li J (2015). Dasatinib enhances antitumor activity of paclitaxel in ovarian cancer through src signaling. Mol Med Rep.

[161] Ma L, Wei J, Su GH, Lin J (2019). Dasatinib can enhance paclitaxel and gemcitabine inhibitory activity in human pancreatic cancer cells. Cancer Biol Ther.

[162] Park NS, Park YK, Yadav AK, Shin YM, Bishop-Bailey D (2021). Anti-growth and pro-apoptotic effects of dasatinib on human oral cancer cells through multi-targeted mechanisms. J Cell Mol Med.

[163] Tian J, Raffa FA, Dai M, Moamer A, Khadang B, Hachim IY, Bakdounes K, Ali S, Jean-Claude B, Lebrun JJ (2018). Dasatinib sensitises triple negative breast cancer cells to chemotherapy by targeting breast cancer stem cells. Br J Cancer.

[164] Morris PG, Rota S, Cadoo K, Zamora S, Patil S, D’Andrea G, Gilewski T, Bromberg J, Dang C, Dickler M, Modi S, Seidman AD, Sklarin N, Norton L, Hudis CA, Fornier MN (2018). Phase II study of Paclitaxel and Dasatinib in metastatic breast Cancer. Clin Breast Cancer.

[165] Nguyen TB, Sakata-Yanagimoto M, Fujisawa M, Nuhat ST (2020). Dasatinib is an effective treatment for angioimmunoblastic T-cell lymphoma. Cancer Res.

[166] Eustace AJ, Crown J, Clynes M, O’Donovan N (2008). Preclinical evaluation of dasatinib, a potent src kinase inhibitor, in melanoma cell lines. J Transl Med.

[167] Jabbour E, Kantarjian H, Ravandi F, Thomas D, Huang X (2015). Combination of hyper-CVAD with ponatinib as first-line therapy for patients with Philadelphia chromosome-positive acute lymphoblastic leukaemia: a single-centre, phase 2 study. Lancet Oncol.

[168] Cortes JE, Kim DW, Pinilla-Ibarz J, le Coutre P (2013). A phase 2 trial of ponatinib in Philadelphia chromosome-positive leukemias. N Engl J Med.

[169] Zhang J, Zhou Q, Gao G, Wang Y, Fang Z (2014). The effects of ponatinib, a multi-targeted tyrosine kinase inhibitor, against human U87 malignant glioblastoma cells. Onco Targets Ther.

[170] Whittle SB, Patel K, Zhang L, Woodfield SE, Du M, Smith V, Zage PE (2016). The novel kinase inhibitor ponatinib is an effective anti-angiogenic agent against neuroblastoma. Invest New Drugs.

[171] Hochhaus A, Gambacorti-Passerini C, Abboud C (2020). Bosutinib for pretreated patients with chronic phase chronic myeloid leukemia: primary results of the phase 4 BYOND study. Leukemia.

[172] Gourd E (2017). Bosutinib more effective than imatinib in CML. Lancet Oncol.

[173] Karim NA, Ullah A, Wang H, Shoukier M, Pulliam S, Khaled A, Patel N, Morris JC (2022). A phase I study of the Non-Receptor kinase inhibitor Bosutinib in Combination with Pemetrexed in patients with selected metastatic solid tumors. Curr Oncol.

[174] Campone M, Bondarenko I, Brincat S, Hotko Y, Munster PN (2012). Phase II study of single-agent bosutinib, a Src/Abl tyrosine kinase inhibitor, in patients with locally advanced or metastatic breast cancer pretreated with chemotherapy. Ann Oncol.

[175] Segrelles C, Contreras D, Navarro EM, Gutiérrez-Muñoz C, García-Escudero R, Paramio JM, Lorz C (2018). Bosutinib inhibits EGFR activation in Head and Neck Cancer. Int J Mol Sci.

[176] Yu L, Guo W, Liu L, Zhang G, Zhang F, Qu Y, Liu Y, Li H, Li H (2019). Bosutinib Acts as a tumor inhibitor via Downregulating Src/NF-κB/Survivin expression in HeLa cells. Anat Rec (Hoboken).

[177] Tan DS, Haaland B, Gan JM, Tham SC, Sinha I, Tan EH, Lim KH, Takano A, Krisna SS, Thu MM, Liew HP, Ullrich A, Lim WT, Chua BT (2014). Bosutinib inhibits migration and invasion via ACK1 in KRAS mutant non-small cell lung cancer. Mol Cancer.

[178] Yun MR, Kim DH, Kim SY, Joo HS, Lee YW, Choi HM, Park CW, Heo SG, Kang HN, Lee SS, Schoenfeld AJ, Drilon A, Kang SG, Shim HS, Hong MH, Cui JJ, Kim HR, Cho BC (2020). Repotrectinib Exhibits Potent Antitumor Activity in Treatment-Naïve and Solvent-Front-Mutant ROS1-Rearranged Non-Small Cell Lung Cancer. Clin Cancer Res.

[179] O’Donohue TJ, Ibáñez G, Coutinho DF, Mauguen A, Siddiquee A, Rosales N, Calder P, Ndengu A, You D, Long M, Roberts SS, Kung AL (2021). Dela Cruz FS. Translational strategies for Repotrectinib in Neuroblastoma. Mol Cancer Ther.

[180] Cervantes-Madrid D, Szydzik J, Lind DE, Borenäs M, Bemark M, Cui J, Palmer RH, Hallberg B (2019). Repotrectinib (TPX-0005), effectively reduces growth of ALK driven neuroblastoma cells. Sci Rep.

[181] Sorensen KD, Borre M, Orntoft TF, Dyrskjot L, Torring N (2008). Chromosomal deletion, promoter hypermethylation and downregulation of FYN in prostate cancer. Int J Cancer.

[182] Alexanian A, Miller B, Chesnik M, Mirza S, Sorokin A (2014). Post-translational regulation of COX2 activity by FYN in prostate cancer cells. Oncotarget.

[183] Garcia S, Dales JP, Charafe-Jauffret E, Carpentier-Meunier S, Andrac-Meyer L, Jacquemier J, Andonian C, Lavaut MN, Allasia C, Bonnier P, Charpin C (2007). Poor prognosis in breast carcinomas correlates with increased expression of targetable CD146 and c-Met and with proteomic basal-like phenotype. Hum Pathol.

[184] Chen ZY, Cai L, Zhu J, Chen M, Chen J, Li ZH, Liu XD, Wang SG, Bie P, Jiang P, Dong JH, Li XW (2011). Fyn requires HnRNPA2B1 and Sam68 to synergistically regulate apoptosis in pancreatic cancer. Carcinogenesis.

[185] Eguchi R, Kubo S, Takeda H, Ohta T, Tabata C, Ogawa H, Nakano T, Fujimori Y (2012). Deficiency of Fyn protein is prerequisite for apoptosis induced by src family kinase inhibitors in human mesothelioma cells. Carcinogenesis.

[186] Berwanger B, Hartmann O, Bergmann E, Bernard S, Nielsen D, Krause M, Kartal A, Flynn D, Wiedemeyer R, Schwab M, Schafer H, Christiansen H, Eilers M (2002). Loss of a FYN-regulated differentiation and growth arrest pathway in advanced stage neuroblastoma. Cancer Cell.

